# Rapid phylogenetic analysis of large samples of recombinant bacterial whole genome sequences using Gubbins

**DOI:** 10.1093/nar/gku1196

**Published:** 2014-11-20

**Authors:** Nicholas J. Croucher, Andrew J. Page, Thomas R. Connor, Aidan J. Delaney, Jacqueline A. Keane, Stephen D. Bentley, Julian Parkhill, Simon R. Harris

**Affiliations:** 1 Pathogen Genomics, The Wellcome Trust Sanger Institute, Wellcome Trust Genome Campus, Hinxton, Cambridge CB10 1SA, UK; 2 Center for Communicable Disease Dynamics, Harvard School of Public Health, 677 Longwood Avenue, Boston, MA 02115, USA; 3 Department of Infectious Disease Epidemiology, Imperial College London, St. Mary's Campus, Norfolk Place, London W2 1PG, UK; 4 Cardiff School of Biosciences, Sir Martin Evans Building, Museum Avenue, Cardiff CF10 3AX, UK; 5 School of Computing, Engineering and Mathematics, University of Brighton, Brighton BN2 4GJ, UK; 6 Department of Medicine, University of Cambridge, Addenbrooke's Hospital, Cambridge CB2 0SP, UK

## Abstract

The emergence of new sequencing technologies has facilitated the use of bacterial whole genome alignments for evolutionary studies and outbreak analyses. These datasets, of increasing size, often include examples of multiple different mechanisms of horizontal sequence transfer resulting in substantial alterations to prokaryotic chromosomes. The impact of these processes demands rapid and flexible approaches able to account for recombination when reconstructing isolates’ recent diversification. Gubbins is an iterative algorithm that uses spatial scanning statistics to identify loci containing elevated densities of base substitutions suggestive of horizontal sequence transfer while concurrently constructing a maximum likelihood phylogeny based on the putative point mutations outside these regions of high sequence diversity. Simulations demonstrate the algorithm generates highly accurate reconstructions under realistically parameterized models of bacterial evolution, and achieves convergence in only a few hours on alignments of hundreds of bacterial genome sequences. Gubbins is appropriate for reconstructing the recent evolutionary history of a variety of haploid genotype alignments, as it makes no assumptions about the underlying mechanism of recombination. The software is freely available for download at github.com/sanger-pathogens/Gubbins, implemented in Python and C and supported on Linux and Mac OS X.

## INTRODUCTION

Many different patterns of genetic variation are evident within important microbial pathogens (1). The simplest cases are the genetically monomorphic, or ‘clonal’, species, such as *Mycobacterium tuberculosis* (2), which typically only accumulate variation through point mutation. Similar patterns of variation are found across much of the genome of species such as *Salmonella enterica* and *Staphylococcus aureus*, with horizontal exchange of sequence usually limited to the movement of mobile genetic elements (MGEs) (3,4). Nevertheless, rare but dramatic recombinations have been recorded outside of MGEs within these species, such as those between *S. enterica* serovars Typhi and Paratyphi A (5) and involved in the generation of the chimeric *S. aureus* sequence type (ST) 239 (6). However, such ‘homologous recombinations’ are far more common in naturally transformable species, such as *Haemophilus influenzae, Neisseria meningitidis* and *Streptococcus pneumoniae* (7). These species possess specific systems that actively import DNA from the environment and subsequently facilitate integration of the acquired fragments into their genome at a region of similar sequence. In such species, diversification often occurs through a complex combination of point mutation, homologous recombination and the movement of MGEs.

The substantial lengths of sequence transferred via different recombination mechanisms can influence genome-wide measures of sequence similarity to a far greater extent than the vertically-inherited point mutations that are the signal of shared common ancestry (8). Hence identifying recombinations, and accounting for their potentially distinctive phylogenetic history, is crucial for tracing the evolution of many important human pathogens. Historically, two approaches have been taken for the detection of recombination events. The first attempts to find ‘exchanges’ within a sample; these methods search for evidence of unexpected similarity between divergent sequences, which are hypothesized to represent the movement of sequence from a donor into a recipient, or a common donor acting as a source of the sequence to both independently. Such exchanges result in homoplasies, which can cause difficulties in identifying the correct phylogenetic topology. One of the first attempts to use such an approach to detect recombination in protein sequences was an investigation of cytochrome evolution (9). Early examples using DNA sequences include tests for ‘incongruent phylogenetic partitions’ (10), distributions of discordant sites (11) and the ‘homoplasy test’ (12). More recent algorithms of this type, such as RDP3 (13), cBrother (14) and GARD (15), are able to identify the breakpoints in putative recombinant sequences based on the relative divergence between multiple representatives of a population in different regions of a sequence alignment.

These methods are most effective when sequences are available for donor, recipient and recombinant genotypes. This is frequently not possible in bacteria owing to the substantial unsampled diversity of potential donors, which may be members of a different species or no longer extant in the population. Hence a second approach, which involves searching closely-related isolates for evidence of ‘imports’ of divergent sequence from a distantly related source, has proved valuable in the analysis of bacterial sequences. Necessarily, such methods are restricted to the study of subsets of bacterial ‘species’ (using the definition of a set of isolates that frequently exchange sequence (16)) as recombinations are identified as regions containing a high density of polymorphisms relative to the background level. One of the first such methods was the application of the maximum *χ*^2^ test to mosaic bacterial genes (17), which identified boundaries between regions of different single nucleotide polymorphism (SNP) density. Such acquisitions of diverse sequence have a dramatic impact on branch length (and hence clock rate estimation), and can also affect tree topology if convergence arises by similar sequences being acquired at the same genomic location in parallel on different branches. The study of recombination in closely-related bacteria led to the concept of a ‘clonal frame’ (18): the portion of a sequence that has not undergone recombination since a set of isolates diverged from their most recent common ancestor. This concept has proved valuable in tracing the evolutionary history of sets of similar bacterial isolates, such as observed in disease outbreaks. The most sophisticated software available to implement this approach is ClonalFrame (19), which uses a Bayesian Monte Carlo Markov chain (MCMC) approach to detect recombinations as regions with a significantly elevated density of polymorphisms while simultaneously constructing a tree based on the point mutations outside of the recombinations. This software has proved popular in the analysis of large collections of genotyping data (20) and smaller collections of whole genome sequences (21).

However, the recent proliferation of second and third generation sequencing technologies has led to a rapid rise in the quantity of bacterial whole genome sequences available for analysis (22), resulting in a significant increase in the computation time taken to fit complex evolutionary models to such rich datasets. Hence, faster algorithms are necessary for the analysis of such samples; one example is BRATNextGen, which detects recombinations, but does not return a phylogenetic reconstruction of the sequences’ past history (23). Here, we present a rapid method for the detection of sequence imports through recombination in bacterial populations. It can be applied to large samples of closely-related haploid sequences in order to generate a prediction of putative recombination events and a phylogeny based on the presumed base substitutions in the ‘clonal frame’. It does not depend upon recombination donors being present in the sample, or require a set of recombination breakpoints to be predefined, a situation for which there is currently a dearth of alternatives capable of processing large genomic datasets (24). The basic statistical framework has already been applied to *S. pneumoniae* (25–30), *Vibrio cholerae* (31), *Mycobacterium abscessus* (32), *S. enterica* serovars Typhimurium (33,34) and Bovismorbificans (35)*, Escherichia coli* (36) and *Neisseria gonorrhoeae* (37), as well as being adapted for an analysis of *Chlamydia trachomatis* (38). This work describes and evaluates a novel implementation in C, called Gubbins (Genealogies Unbiased By recomBinations In Nucleotide Sequences), which represents the first distribution of this software and improves on all previous applications of this type of method through using joint (rather than marginal) ancestral sequence reconstructions, allowing for different strategies for phylogeny construction, and identifying recombinations at speeds that make it applicable to collections of hundreds or thousands of bacterial genomes. This work describes how simulated datasets indicate this algorithm accurately identifies recombination and significantly improves the estimation of phylogenetic topologies and branch lengths. It rapidly converges on these results both using alignments of artificial, complete or draft genome sequences. This software is distributed to be used on Linux and Mac OS X systems and can be freely downloaded from github.com/sanger-pathogens/Gubbins.

## MATERIALS AND METHODS

### Description of algorithm

The algorithm first detects polymorphic sites in the input alignment, which are used to generate a maximum likelihood phylogeny using either RAxML (39) or FastTree 2 (40), in each case using a general time-reversible (GTR) model of substitution and a gamma distribution to account for rate heterogeneity between sites. A joint ancestral sequence reconstruction of base substitutions is then performed based on the alignment of polymorphic sites, tree topology and branch lengths using FastML (41), again using a GTR substitution model. Optionally, a Gamma distribution of rates can also be used in this reconstruction. Positions are considered to be undetermined at internal nodes in instances where the equivalent positions are ambiguous in all of the downstream leaf node sequences. This processing defines both the base substitutions occurring on each branch of the phylogeny and the distances between them in terms of the number of identified bases on the branch (i.e. excluding bases that were undetermined on the branch).

A scan statistic was selected as a flexible, non-parametric sliding window tool for identifying clusters of base substitutions, which are presumed to represent a point process (42). A scan is performed on each branch independently, initially conditioned on the number of substitutions occurring on the branch, *s*_B_. The null hypothesis for branch B, *H*_0,B_, assumes that base substitutions arise through point mutation randomly across the genome. In principle, as with all methods that rely on detecting recombinations as regions of heightened base substitution density, the method is therefore susceptible to false positive inferences of recombination if mutations accumulate at substantially different rates across the genome (43). Only relatively small heterogeneities in point mutation rates have been observed across bacterial genomes (44), but these can become significant over time, as can the effect of varying selection pressures at different loci. Hence, the regions of elevated diversity detected by this algorithm may represent mutational ‘hotspots’, or regions under relaxed or positive selection, on branches representing long periods of divergence. Furthermore, while Gubbins could be applied to alignments of individual loci, rather than whole genomes, the sliding window approach requires that recombinations are short relative to the length of the alignment.

The null hypothesis, *H*_0,B_, is modeled such that the number of base substitutions occurring in a window, *s_w_*, follows a binomial distribution based on the number of bases in a window, *w*, and the mean density of base substitutions across the number of bases called on the downstream node (i.e. the genome length, *g*, corrected for missing data), *d*_0,B_*.* The value of *w* is altered between 0.1 and 10 kb such that the expected number of base substitutions in a window would be at least 10 according to *H*_0,B_. Shorter windows allow for more precise identification of recombination breakpoints when *s*_B_ is high, but lack power to detect longer recombinations introducing a relatively low density of base substitutions when *s*_B_ is small. On each branch B, *s*_B_ windows are tested, each centered on a different base substitution. Hence the threshold *P*-value (*P*_threshold_) is a Bonferroni corrected (45) value of $\frac{{0.05}}{{s_{\rm B} }}$.

Each putative recombination, r, is identified as a set of contiguous sliding windows, each containing *s*_w_ base substitutions at an elevated density such that *H*_0,B_ can be rejected at *P*_threshold_ using a one-tailed Binomial test, based on the likelihood that at least *s*_w_ base substitutions would be observed in a locus of length *w*. This block r is proposed to conform to an alternative hypothesis, *H*_1,B,r_, that is also modeled as a binomial distribution based on its length, *l*_r_, and a higher density of substitutions, *d*_1,B,r_, calculated from *l*_r_ and the number of base substitutions within r, *s*_r_. The length of r is then reduced such that its boundaries are the sites of the outermost base substitutions it encompasses. Each end of the block is then progressively moved inward to the next base substitution position using a trimming process which is alternately carried out on the left, then right, side of r. Trimming ceases when further contraction of r on either side no longer increases its likelihood under *H*_1,B,r_ relative to its likelihood under *H*_0,B_. Lastly, an inequality has to be satisfied as a test for rejection of *H*_0,B_ based on the final trimmed length of the recombination, *l*_f_, and the remaining number of base substitutions it contains, *s*_f_:

\begin{equation*} \frac{{0.05}}{{g/l_{\rm f} }} {>} 1 - \sum\limits_{i = 0}^{i = s_{\rm f} - 1} {\left( {\begin{array}{*{20}c} {l_{\rm f} } \\ i \\ \end{array}} \right)(d_{0,{\rm B}} )^i (1 - d_{0,{\rm B}} )^{l_{\rm f} - i} } \end{equation*}

The left side of the inequality represents a threshold probability with a Bonferroni correction (45) for the number of similarly-sized non-overlapping windows that could be observed in the genome. The right side calculates the probability, under *H*_0,B_, that such a window would contain at least *s*_f_ base substitutions. This condition is required to eliminate false positive ‘recombination’ identifications that span two distinct clusters of base substitutions. By definition, such events have few base substitutions in their center, and therefore after trimming of the initial block may no longer contain a significantly elevated density of base substitutions.

Of the set of blocks identified on B in this manner, that having the smallest likelihood ratio (r_smallest_), calculated as the probability of the block under *H*_0__,B_ divided by its probability under *H*_1__,B,r_, is identified. The span of sequence encompassed by r_smallest_ is then converted to ‘missing data’ on branch B and all descendants, as the recombination overwrites any information from the ‘clonal frame’. The *d*_0,B_ is then recalculated based on the mean density of substitutions across the remainder of the chromosome outside of r_smallest_. The identification of recombinations occurring on B is then repeated, with the process iterating until either no more loci deviate from *H*_0__,B_ or the minimum number of substitutions within a window required to identify a recombination falls below the threshold minimum number of substitutions required to detect a recombination (*s*_min_; set to three by default). This process is repeated for each branch on which more than *s*_min_ base substitutions are reconstructed as having occurred.

Following the masking of putative recombination events by replacing such sequence with missing data, a second maximum likelihood phylogeny and sequence reconstruction are produced using the specified phylogenetic software and FastML, respectively. This second tree will therefore not be influenced by the base substitutions occurring within detected recombinations, which should result in a more accurate ancestral sequence reconstruction. The algorithm then performs multiple iterations until a pre-specified maximum threshold is reached (set to five by default), unless convergence occurs first. Convergence is assessed through two iterations, not necessarily consecutive, appearing identical based on a pre-specified test. Multiple options for tests are implemented in the software: the occurrence of identical patterns of recombination, identical tree topologies or identical trees (taking into account both topology and branch lengths). In the case of the occurrence of identical patterns of recombination or identical trees, the algorithm will have reached a point at which any further iterations would simply recapitulate earlier iterations. When identical tree topologies occur, this is not necessarily the case. In the analyses described in the ‘Results’ section, convergence was assessed through the occurrence of identical trees, with a maximum of five iterations performed unless otherwise specified.

### Simulation of artificial sequences

Alignments were generated by discrete step, forward time individual-based birth-death simulations of a pneumococcal lineage based on a previously described approach (46). The ancestral genotype was that of *S. pneumoniae* ATCC 700669 [EMBL accession code: FM211187] (47), the reference isolate for the PMEN1 lineage. For the simulation of recombination within the PMEN1 lineage, the whole genome alignment described in (26) was used as the set of potential donors. For the analysis of exchange with other pneumococci, 100 nt paired end reads were simulated from the sequences listed in Supplementary Table S1 and mapped against the ancestral genotype as described previously (48) to generate the aligned donor sequences.

In each simulation, all extant sequences acquired a single point mutation in each timestep from *t* = 0 to *t* = *t*_max_. Each point mutation corresponded to a single base substitution at a random position using a Jukes-Cantor substitution model. Sequences also underwent recombination with a probability of *p*_rec_ at each timestep. Each recombination used a randomly selected donor from the appropriate alignment and a random starting position, with extension of the exchange stopping with a per-base probability of 0.00016 bp^−1^; this generated the geometric distribution of homologous recombination lengths observed in the pneumococcus (48). Where recombination was permitted to occur within the lineage, at each timestep the set of potential sequence donors consisted of those listed in Supplementary Table S1, plus an equally numerous set of randomly-selected sequences extant in the simulated population; recombinations were therefore equally likely to arise from within the lineage, or from a sequence in Supplementary Table S1.

Furthermore, each sequence had a probability *p*_birth_ of being duplicated into two independently diversifying sequences, corresponding to a bifurcation event in the phylogeny. Each sequence also had a probability *p*_death_ of being sampled at each timestep, after which point it no longer diversified or served as the progenitor to any other sequences. The simulation was stopped at *t*_max_ once the total number of sampled and extant sequences in the simulation reached (or exceeded) a pre-determined maximum, *n*_seq_, of 100 unless otherwise specified. The ratio of *p*_death_ to *p*_birth_ was held constant at 0.75 over all simulations.

When comparing the output of Gubbins to the input simulation, only substitutions that had defined ancestral and derived alleles were analyzed (i.e. substitutions that involved missing data were ignored). Substitutions were counted as ‘true positives’ where the correct change of one base to another was reconstructed as having occurred in the matching set of isolates. They were deemed to have been assigned to a recombination if they lay between its boundaries and occurred on the same branch of the phylogeny. The ancestral sequence of *S. pneumoniae* ATCC 700669 was included in the alignments, and used as the outgroup on which to root each phylogeny produced by Gubbins to allow for the accurate calculation of the correlation between root-to-tip distance and date of sampling.

### Analysis of whole genome data

Analysis of the full set of PMEN1 sequences used the alignment from (26); 11 closely-related isolates were extracted as a subsample for comparison with the output of ClonalFrame. For the analysis of *S. aureus* ST239, 14 representatives from the South-East Asian clade were extracted from the larger alignment (49) for the equivalent comparative analysis. For the analysis of *Helicobacter pylori*, eight publically available complete genomes were selected from across the species that included both the most closely-related pair of isolates and the isolate most divergent from the rest of the sample, based on a previous analysis (50). These genomes were then aligned using progressiveMauve (51), generating a 1.8 Mb core genome alignment for analysis.

The resulting whole genome alignments were then analyzed using the default settings of Gubbins, except that the *S. pneumoniae* and *S. aureus* analyses were run until convergence. For *S. pneumoniae* and *S. aureus*, ClonalFrame (19) was also run using default settings, without estimating node ages, with a burn in chain length of 25 000 and a parameter estimation chain length of 25 000. For *H. pylori*, convergence was achieved when ClonalFrame was run without estimating node ages or theta, using a burn in chain length of 10 000 and a parameter estimation chain length of 10 000. Convergence was assessed through plotting the variation in parameter values over the course of the MCMC; these are shown in Supplementary Figures S4, S7 and S9.

## RESULTS

### Gubbins produces accurate reconstructions

To assess the accuracy of Gubbins in reconstructing the evolutionary history of bacterial samples, divergence of sequences from a single common ancestor (*S. pneumoniae* ATCC 700669) through point mutation and homologous recombination was simulated using a discrete timestep birth-death process. The sequence donors for the homologous recombinations were other publicly available complete pneumococcal genomes (Supplementary Table S1), all of which are substantially divergent from the recipient genotype (52). Ten simulations were performed at each of eight different relative rates of homologous recombination between a per time step probability of recombination (*p*_rec_) of 0.01, representing near clonal evolution, and 0.75, where homologous recombinations were three-quarters as frequent as point mutations. The output whole genome alignments from these simulations were then analyzed using Gubbins. In each output of Gubbins, all base substitutions in the reconstructions were assumed to either originate as polymorphisms introduced through recombination, or else represent ‘point mutations’. The accuracy of the reconstructions was assessed through positive predictive values (PPVs), the proportion of true positives (see Materials and Methods) in the results of Gubbins; the false discovery rate (FDR), the proportion of false positives in the results of Gubbins; and the false negative rate (FNR), the proportion of base substitutions that occurred in the simulation that were not correctly identified by Gubbins.

**Figure 1.**
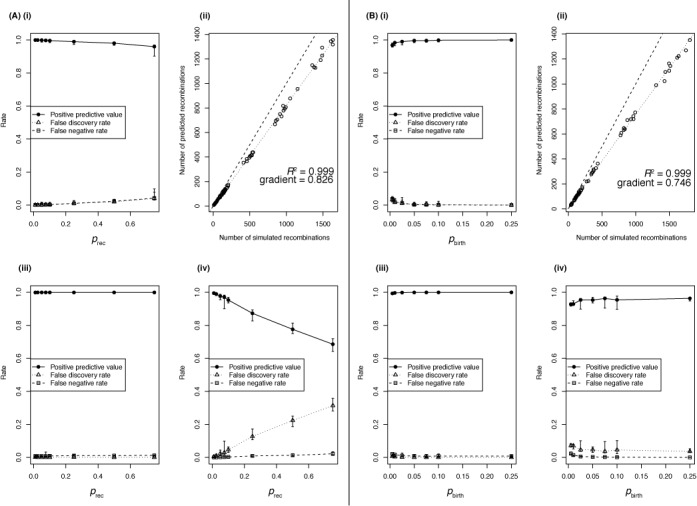
Accuracy of Gubbins reconstructions from simulations using diverse sequences as recombination donors. (**A**) Impact of changing the rate of recombination (*p*_rec_) relative to the rate of point mutation on the accuracy of Gubbins’ evolutionary reconstructions. (i) The accuracy of the overall reconstructed set of substitutions; each datapoint represents the median of 10 simulations, with the error bars representing the full range of values. (ii) The relationship between the number of simulated recombinations and the number of recombinations identified by Gubbins across the full dataset represented in (i). The dashed line represents the identity line and the dotted line is the best fit to the data; the gradient and Pearson correlation coefficient of the best fit line are annotated on this graph. (iii) The accuracy with which the correctly identified base substitutions were assigned as occurring through recombination rather than through point mutation. Metrics are plotted as in (i). (iv) The accuracy with which correctly identified base substitutions were identified as point mutations. Metrics are again represented as in (i). (**B**) Impact of changing the level of diversification (*p*_birth_) between sequences being sampled from the simulated dataset. Plots again represent the output of 10 simulations for each value of *p*_birth_, and show the same statistics as described in (A).

Across all simulations, the overall FastML reconstruction of base substitutions from the final iteration was very accurate, with all PPVs above 90% and all FDRs and FNRs below 10% (Figure 1A(i)). The linear relationship between the number of simulated and detected recombinations (*R*^2^ = 0.999) across simulations indicates that the sensitivity (around 83%) for the detection of recombinations did not vary dramatically as *p*_rec_ was altered (Figure 1A(ii)). The high sensitivity of the algorithm likely reflects its design to avoid the occurrence of one recombination inhibiting the detection of others occurring on the same branch (see ‘Materials and Methods’). Those recombinations that were not detected generally had little impact on the sequences’ divergence. Eleven per cent of the recombinations could not be identified because they caused two or fewer base substitutions, and therefore were not able to meet the criterion of *s*_min_, the minimum number of base substitutions required to infer a recombination, being set to three by default.

The *s*_min_ parameter is one reason for Gubbins’ high specificity, an important factor in the analysis of clonally evolving samples where false positives may be biologically misleading. Of the correctly identified base substitutions, few point mutations were incorrectly inferred as being caused by recombinations; hence, the PPVs for the identification of base substitutions introduced by recombination were all greater than 99.5% (Figure 1A(iii)). The corresponding FNRs were low, peaking at just over 3%; these represented the substitutions introduced by undetected recombinations being misclassified as point mutations. Hence, these same substitutions can also be regarded as false positive point mutations; as the true positive number of point mutations is much lower than the number of base substitutions imported by recombination, the point mutation FDR is greater in magnitude (Figure 1A(iv)). The linear increase in this FDR with *p*_rec_ reflects the constant proportion of recombinations that were undetected across this set of simulations, whereas the expected number of point mutations per branch remained constant. Nevertheless, the PPVs for point mutations were around two-thirds or greater even at *p*_rec_ values of 0.75, despite all 10 simulations using this parameterization having at least 35-fold more base substitutions introduced through recombination than occurring by point mutation.

### The impact of sequence divergence levels

To investigate the effect of changing the timescales of diversification between sequences, a further set of simulations were carried out with different values of *p*_birth_, the parameter controlling the rate at which the tree bifurcates. The PPVs for the reconstruction of base substitutions were above 95% across all simulations, increasing as samples became more closely related (i.e. high *p*_birth_; Figure 1B(i)). Again, the number of predicted recombinations increased linearly with the number introduced in all simulations (*R*^2^ = 0.999; Figure 1B(ii)). The approximate sensitivity was slightly lower in this case (75%) than when *p*_rec_ varied, because branches containing high numbers of recombinations were also associated with high numbers of point mutations, thereby impacting on the statistical power to detect recombinations based on an elevated density of base substitutions above the background level. Gubbins retained its high levels of specificity and sensitivity: PPVs for the base substitutions reconstructed as being imported by recombination were all above 99%, and FNRs all 2.6% or lower (Figure 1B(iii)). This was also reflected by the PPVs for point mutations all being above 89% (Figure 1B(iv)), with no linear increase in FDR with variation in *p*_birth_ as the number of true positive point mutations rose in proportion to the number of recombinations, the source of false positives. However, there are signs of a non-linear rise in the point mutation FDR at very low values of *p*_birth_, approaching the extreme situation in which recombinations cannot be detected due to the expected density of point mutations rising toward the density at which base substitutions are imported by recombination.

Two scenarios in which the algorithm's assumptions were challenged were similarly tested. In the first, recombinations could either represent imports from the same set of donors as in the previous simulations, or correspond to exchanges within the simulated lineage. These two events occurred with equal probability, summing to *p*_rec_ (Supplementary Figure S1). As expected, the approximate sensitivity of the algorithm was almost halved (48%) when *p*_rec_ was varied, as 49% of the recombinations imported fewer than *s*_min_ SNPs; these are likely to correspond mainly to the exchanges between members of the simulated lineage. However, as these recombinations had little effect on the recipient sequences, the PPV of base substitutions assigned as arising through recombination remained very high when either *p*_rec_ or *p*_birth_ was altered. Correspondingly, the FDRs and FNRs were low and similar to those observed in Figure 1. Hence, when exchanges are common within the sample, Gubbins’ prediction of recombination again remains specific, but as with any algorithm using the spatial distribution of base substitutions to detect horizontal transfers of sequence, it suffers a loss of sensitivity.

In the second scenario, sequences very similar to the starting genome, *S. pneumoniae* ATCC 700669, were used as the recombination donors in a further set of simulations (Supplementary Figure S2). The overall reconstruction of base substitutions remained highly accurate, and the identification of base substitutions arising from recombinations was still very specific. However, Gubbins was only able to predict 5–10% of the actual number of recombinations, largely as a consequence of only 35% of the recombinations importing more than *s*_min_ base substitutions. The FNRs for the base substitutions resulting from recombinations were higher than in the previous set of simulations, as the absolute numbers of true positive base substitutions arising from recombination were low in this scenario. Yet, the PPVs for point mutations were very similar to those in the previous set of simulations, as they occurred at the same frequency independent of the nature of the recombination sequence donor. Therefore, when recombinations originate from donors closely related to the recipient, Gubbins will highlight regions of imported divergent sequence while still generating an accurate phylogenetic reconstruction.

### Gubbins improves phylogenetic accuracy

**Figure 2.**
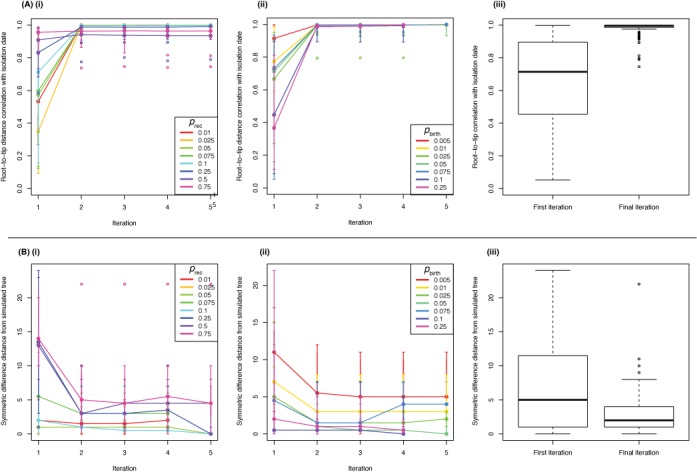
Accuracy of phylogenetic reconstructions from Gubbins analyses. (**A**) Accuracy of branch length estimation. (i) For each simulated dataset represented in Figure 1A, the Pearson correlation (*R*^2^) of the root-to-tip distances of simulated sequences with the number of time steps over which they had been diverging from the original sequence was calculated for the phylogenies from each iteration of the Gubbins analyses. The solid points linked by lines represent the median of the 10 simulations for each parameter set at each iteration; the vertical bars indicate the most extreme datapoints within 150% of the interquartile range. Empty circled points indicate outliers beyond this boundary. The color of each line indicates the *p*_rec_ parameter value in the simulations. (ii) The same statistics were calculated for the simulations displayed in Figure 1B. The results are displayed as in panel (i), with the color of the line representing the *p*_birth_ parameter value used in the simulation. (iii) Box and whisker plot summary of the *R*^2^ values for all simulations represented in Figure 1 for the ‘naïve’ phylogenies in the first iteration and the phylogenies from the final iteration. The whiskers extend to the most extreme datapoints within 150% of the interquartile range, with empty circled points representing outliers. (**B**) Accuracy of tree topologies. (i) For each simulated dataset represented in Figure 1A, the symmetric differences in terms of branching patterns between the actual history with which the simulated sequences diverged and the reconstructed phylogeny topologies from each iteration of the Gubbins were calculated. The median distances, and associated variation, are plotted for each *p*_rec_ value as in panel (A) (i). (ii) The same distances are calculated from the simulations displayed in Figure 1B, with the color of the line representing the *p*_birth_ parameter value used in the simulation. (iii) Boxplot summary of the symmetric differences for all simulations represented in Figure 1 for the ‘naïve’ phylogenies in the first iteration and the phylogenies from the final iteration, displayed as described in panel (A) (iii).

Gubbins is designed to run iteratively, although by default it stops after five iterations unless identical phylogenies are identified in two iterations, which indicates convergence has occurred. Two metrics were used to compare the maximum likelihood phylogenies from each of the iterations of the analyses presented in Figure 1. The first was to assess changes in branch length through calculating the correlation between root-to-tip distance of each leaf node in the phylogeny with the simulation timestep at which its diversification was halted (Figure 2A), which should be exactly linear in the case of the simulated strict molecular clock. Both when varying *p*_rec_ and *p*_birth_, the initial broad spread of *R*^2^ coefficients (median of 0.714) from the ‘naïve’ initial maximum likelihood tree rapidly improved by the second iteration, at which point Gubbins had made its first prediction of recombinations. There was little refinement afterward, with a final median *R*^2^ of 0.996 (Figure 2A(iii)). Overall, the correlation values were improved in 96% of simulations, with the exceptions typically representing reconstructions confounded by high *p*_rec_ values.

The topology of the trees generated by each iteration of the Gubbins analyses was also compared with that representing the true pattern of divergence from the simulation via symmetric differences between the branching patterns (53) (Figure 2B). Again, a broad spread of values from the naïve maximum likelihood phylogenies (median = 5) fell to a narrower range of generally lower values by the final iteration (median = 2). The starting topology's accuracy was maintained or improved in 99% of cases. Lower levels of accuracy were again associated with simulations using extreme *p*_rec_ and low *p*_birth_ values. It might be expected that the simulations in which exchange between sequences within the lineage occurred would contain a greater number of topological errors in the final phylogeny, as such recombinations can often result in homoplasies. However, similar analysis of these simulations (Supplementary Figure S3) revealed that the starting topology's accuracy was again maintained or improved in 99% of cases, with the distribution of final symmetric differences similar to the set of simulations in which no exchange occurred (median = 2). In neither set of simulations was there evidence that extending runs further would improve the results, indicating that convergence had effectively been achieved after a small number of iterations.­

### Gubbins rapidly converges on large datasets

**Figure 3.**
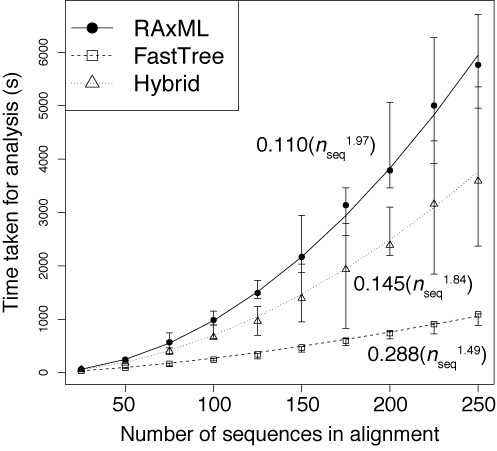
Relationship between Gubbins analysis times and number of sequences in alignment (*n*_seq_). The time taken for the analysis of alignments containing different numbers of simulated sequences are shown when using RAxML for constructing the phylogeny at each iteration (solid line), using FastTree 2 for constructing the phylogeny at each iteration (dashed line), or a hybrid approach that uses FastTree 2 in the first iteration and RAxML for all subsequent iterations (dotted line). The formulae for each of the best fit trend lines are displayed.

This rapid convergence on an accurate solution is crucial in the analysis of large datasets. To quantify the time required for Gubbins analyses, 10 sets of 250 *S. pneumoniae* sequences were simulated under consistent parameter settings of *p*_rec_ = 0.1 and *p*_birth_ = 0.05. These were then randomly subsampled to generate sets of alignments with differing numbers of sequences (*n*_seq_) separated by a similar level of divergence (Figure 3), which were used to benchmark the algorithm with a set of HP ProLiant BL460c G6 machines with Intel X5650 2.6 Ghz processors. The dependence of the run time on *n*_seq_ was found to be quadratic, best described by the curve 0.110(*n*_seq_^1.97^). This was achieved using phylogenies constructed with RAxML (39), a rapid and accurate maximum likelihood algorithm for generating phylogenies. However, much of the analysis time is dedicated to constructing the initial phylogeny, which integrates the information from all polymorphic sites across the alignment before any recombinations are first identified. Therefore, run times were also recorded for analysis of the same alignments with a ‘hybrid’ approach that used FastTree 2 (40), an algorithm that quickly calculates approximately maximum likelihood phylogenies, for tree construction in the first iteration, and RAxML in subsequent iterations. This reduced both absolute run times and the rate at which the times increased with larger numbers of sequences in the alignment. Gubbins can also use FastTree 2 in every iteration, which reduced the dependence of run time on the number of sequences in the alignment to 0.341(*n*_seq_^1.45^), and increased their consistency.

**Figure 4.**
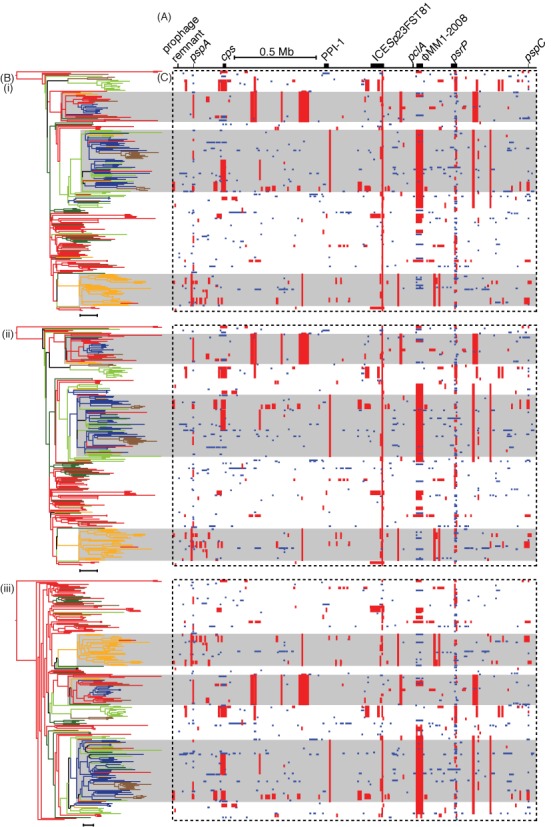
Analysis of the PMEN1 genome alignment with Gubbins employing different phylogeny construction strategies. (**A**) The simplified annotation of the *S. pneumoniae* ATCC 700669 genome. (**B**) The maximum likelihood phylogenies generated from the whole genome alignment of 241 *S. pneumoniae* PMEN1 isolates after re-analysis using the Gubbins algorithm either relying on (i) RAxML for constructing the phylogeny in each iteration, (ii) the hybrid approach of FastTree 2 for the first iteration and RAxML for subsequent iterations, or (iii) FastTree 2 for constructing the phylogeny in each iteration. Each phylogeny was midpoint rooted and colored according to location, as reconstructed through the tree from the countries of isolation of the sequences using maximum parsimony: red for Western Europe, brown for Eastern Europe, light green for North America, dark green for South America, yellow for South Africa and dark blue for South-East Asia. The backgrounds of three large clades are shaded gray to aid the alignment of the phylogeny with the panels in (C) (i–iii). Scale bars underneath the phylogenies represent a phylogenetic distance of 20 point mutations. (**C**) These panels represent the pattern of predicted recombinations from the analyses using the three different phylogeny estimation approaches (i–iii). Each column relates to a base in the reference genome; each row represents an isolate in the phylogeny. Red blocks indicate predicted recombinations occurring on an internal branch, which are therefore shared by multiple isolates through common descent. Blue blocks represent recombinations that occur on terminal branches, which are unique to individual isolates.

These same three approaches were also each applied to an alignment of 241 *S. pneumoniae* PMEN1 sequences, representing a previously analyzed dataset with the distantly-related outgroup removed (26). All of the major features of the previously reported analysis were recapitulated (Figure 4), with the most noticeable difference being that RAxML and FastTree 2 differed in their placing of the longest branch on which the tree was midpoint rooted in these analyses. Analysis with RAxML alone took 8.39 h; with the hybrid approach, it took 3.39 h; and with FastTree 2 used in all iterations, analysis completed in 32.0 min. These times were all significantly longer than those taken to analyze the simulated alignments of similar size, which likely reflects the greater extent of missing data in the PMEN1 alignment resulting from the draft nature of the genomes (as opposed to the complete genomes used in the simulations) and variation in MGEs, a process not included in the simulations.

### Comparison with ClonalFrame

**Figure 5.**
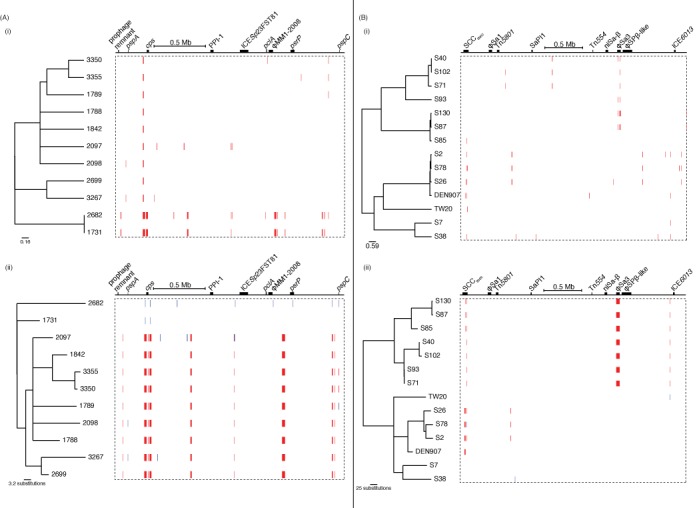
Comparison of ClonalFrame and Gubbins analyses of *S. pneumoniae* and *S. aureus* sequences. (**A**) Analysis of 11 *S. pneumoniae* PMEN1 sequences using (i) ClonalFrame and (ii) Gubbins. (**B**) Analysis of 14 *S. aureus* ST239 isolates using (i) ClonalFrame and (ii) Gubbins. The output of Gubbins is displayed as described in Figure 4. The output of ClonalFrame is displayed in a similar manner, with the tree representing the 50% majority-rule consensus phylogeny, and the red blocks indicating recombinations defined by a contiguous set of sites with a posterior probability of recombination above 0.5, with at least one site having a posterior probability above 0.95, as described in (55).

Given Gubbins’ apparent accuracy based on simulations, it was compared to ClonalFrame using draft genomic data. A sample of 11 *S. pneumoniae* sequences, corresponding to serotype 19A vaccine escape isolates from the USA distinguished by a total of 1516 polymorphic sites, were taken from the alignment generated in (26). Analysis with Gubbins converged on the sixth iteration, as assessed through the occurrence of identical phylogenies in two iterations. The run took a total of 48.4 s on an HP ProLiant DL385 G2 Linux machine with an AMD Opteron 2218 2.60 Ghz processor, and predicted 28 recombination events. Using the tests for the presence of recombination implemented in PhiPack (54), it was found that both the spatial distribution of polymorphic sites (maximum *χ*^2^ test *P*-value of 0.00) and presence of homoplasies (pairwise homoplasy index, or PHI, *P*-value of 3.94×10^−36^) suggested recombination was present in the dataset. Following the removal of the recombinations predicted by Gubbins from the appropriate sequences, neither test found significant evidence of recombination in the final alignment (maximum *χ*^2^ test *P*-value of 0.92; PHI *P*-value of 0.08). ClonalFrame was run on the same input (therefore without removing accessory genomic loci as is recommended for this software (21)) with 25 000 iterations of the chain removed as burn-in, and a further 25 000 iterations used for parameter estimation. This analysis took 705.4 h on the same machine, and appeared to have effectively converged based on the variation in parameter estimates during the MCMC chain, although there is notable bistability in some parameters (Supplementary Figure S4). This analysis predicted 48 recombination events, using the criteria of a set of contiguous sites with a posterior recombination probability above 0.5 where at least one of the sites has a posterior recombination probability exceeding 0.95 (55) (Figure 5A). In the absence of an outgroup, the directionality of recombination cannot be accurately determined by either method, and therefore events shared by complementary sets of leaf nodes can be considered equivalent.

The results of both algorithms were similar, with some instructive small differences. For instance, the output of ClonalFrame suggested that isolates 2682 and 1731 are very closely related, with essentially all of the divergence between them attributed to recombination, whereas Gubbins reconstructed 339 substitutions distinguishing the pair as having arisen through recombination, with 35 scattered base substitutions predicted to have arisen as point mutations. The only significant difference between the tree topologies is that isolate 1789 is the sister group to the clade formed by 3350 and 3355 in the ClonalFrame output, whereas 1842 occupies the equivalent position in the Gubbins analysis. Of the 38 polymorphic sites that were fully defined in each of these four isolates, 19 supported the partition generated by Gubbins and 11 supported the partition suggested by ClonalFrame. Two of these sites supporting the ClonalFrame partition were found within the *pspC* gene; in the Gubbins reconstruction, these were instead independently acquired through similar recombinations occurring in parallel on different branches of the tree. This is biologically plausible, as *pspC* is a highly variable and mosaic gene, which encodes an antigen likely to be under diversifying selection imposed by the host immune system (56). By contrast, the ClonalFrame analysis suggested 1789, 3350 and 3355 all shared a recombination through common descent; however, this did not span any sites that were polymorphic in these three isolates or 1842. The inclusion of such ‘silent’ recombinations in the ClonalFrame reconstruction is in direct contrast to the output of Gubbins, in which each recombination has to be supported by at least three base substitutions; this constraint likely accounts for the more irregular distribution of recombination lengths from Gubbins, whereas those predicted by ClonalFrame more closely match the prior expectation of a geometric length distribution (Supplementary Figure S5).

Gubbins and ClonalFrame were also both applied to the core genome alignment of eight *H. pylori* sequences, which represented much of the observed diversity within the species (50). In such datasets, the identification of recombinations as regions with elevated densities of base substitutions is confounded by the high diversity of the sequences in the alignment, and therefore for improved accuracy such populations would need to be split into sets of closely-related isolates. Nevertheless, both Gubbins and ClonalFrame produce similar results to a previous analysis, only disagreeing on the topology at the base of the tree (Supplementary Figures S6 and S7). The main difference was that ClonalFrame predicted a larger number of longer recombinations (Supplementary Figure S5), with Gubbins’ identification of recombinant sequence being more conservative.

### Analysis of clonal evolution

*Staphylococcus aureus* is considered to be a predominantly clonally-evolving species, based on the rarity with which MLST loci were observed to be exchanged between representatives (57) and the low level of homoplasy in the *S. aureus* ST239 phylogeny (4). A sample of 14 *S. aureus* ST239 isolates, distinguished by 1259 polymorphic sites, were also analyzed using both Gubbins and ClonalFrame (Figure 5B). While the PHI test confirmed there was little homoplasy in the starting alignment (*P-*value of 0.39), evidence was found of imported sequence based on the maximum *χ*^2^ test (*P-*value of 0.00). Analysis with Gubbins converged on the fourth iteration, taking 46.4 s and predicting 11 recombinations using the same machine as for the PMEN1 analyses. Following the removal of these 11 putative recombinations, the maximum *χ*^2^ test no longer indicated the presence of further recombination in the dataset (*P-*value of 0.75). Six predicted recombinations occurred in the SCC_mec_, the variable genomic island associated with beta lactam resistance. Another extended to almost the entire length of the 44.7 kb prophage φSa3 (58), with its edges just 680 and 96 bp within the respective 5′ and 3′ boundaries of the annotated MGE. Comparative genomic analysis confirms this recombination distinguishes two sets of isolates carrying different prophage at the φSa3 insertion site, although the viral sequence appears to have been deleted in S71 (Supplementary Figure S8). Two further recombinations had identical boundaries to one another, suggesting they might represent a recurring atypical change in the genome; that left only two other putative homologous recombinations in the dataset. ClonalFrame, again run with 25 000 burn-in generations and 25 000 parameter estimating generations, completed in 861.2 h, with a more robust level of convergence than observed with the *S. pneumoniae* analysis (Supplementary Figure S9). The output predicted 36 recombinations. Ten were found in φSa3, but did not match the observed change in prophage, and seven occurred in the SCC_mec_ island. The remaining 19 recombinations did not occur within annotated genomic islands; three of these overlapped with the two putative homologous recombinations identified by Gubbins. Almost all of the recombinations predicted by ClonalFrame were very short, which likely reflects ClonalFrame's prior expectation of a geometric distribution of recombination lengths (Supplementary Figure S5).

One notable difference in the tree topologies was that in the phylogeny from ClonalFrame, S93 was an outgroup to the clade of S71, S40 and S102, whereas in the output of Gubbins, the former pair and latter pair both formed separate clades. Of the 59 polymorphic sites at which bases were called in all four isolates, 47 supported the partition identified by Gubbins, which did not attribute any of them to having arisen through recombination. By contrast, ClonalFrame predicted six recombinations with high posterior probabilities within this clade, but these only encompassed one polymorphic site that varied across the four sequences. There was also a notable difference in the lengths of the branches connecting the very similar pair S87 and S130 with S85. Gubbins indicated they had diverged over a considerable period, corresponding with the input alignment showing there are 81 fully defined SNPs that distinguish S85 from S87 and S130, which are themselves only distinguished by only seven polymorphic sites. The output of ClonalFrame indicated that the common ancestor of all three isolates only shortly predated the common ancestor of S87 and S130, and predicted eight recombinations with high posterior probabilities within the clade. However, these only accounted for two of the polymorphic sites distinguishing the sequences, suggesting other factors within ClonalFrame's model informed the phylogeny.

## DISCUSSION

Gubbins is a fast, accurate and flexible algorithm that detects regions of elevated sequence diversity in large bacterial genomic datasets and removes them from alignments in order to increase the accuracy of phylogenetic reconstructions. Simulations indicate that the algorithm is most effective when detecting imports of sequence into a densely sampled collection of closely-related isolates, where recombinations import a high density of base substitutions from divergent donors. In scenarios where these conditions do not apply, the method remains specific in its detection of recombination, and still improves the accuracy of phylogenetic reconstructions. As with all similar methods, sensitivity is lower for the detection of recombinations that import few base substitutions or are genetically ‘silent’, but these have relatively little impact on the structure of the inferred genealogy or the overall contribution of horizontal sequence exchange to a lineage's evolution.

Gubbins’ specificity makes it applicable to clonally-evolving bacteria with a low risk of false positive detections of recombination, and very few samples would be expected to reach the extremely high *p*_rec_ values at which accuracy decreased (20). For instance, the analysis of the highly recombinogenic *S. pneumoniae* PMEN1 lineage (26) required detection of sequence imported from other diverse pneumococci, with an inferred *p*_rec_ of 0.10 and *p*_birth_ of 0.069, both within the range of parameters over which Gubbins has a low error rate. Inaccuracies that may arise as a consequence of high levels of divergence between isolates in an alignment can be overcome by denser sampling or subdivision of the population prior to analysis (28,30), though in common with all phylogenetic methods, this will ultimately reach a limit of resolution beyond which no further information on the lineage's diversification can be obtained through sequence analysis.

Gubbins is also a practical tool in the context of current sequencing capacity; it is typically able to converge on a result for an alignment of 100 two megabase sequences in well under an hour, whereas the sophisticated model fitting of ClonalFrame takes weeks to analyze a much smaller number of genomes. Furthermore, the algorithm's flexibility allows it to be applied to full genome alignments without the difficult process of filtering to remove accessory loci, as is recommended for ClonalFrame. This is clear from the predicted recombinations affecting the φSa3 prophage in *S. aureus*; while the wholesale replacement of the viral sequence is accurately represented by Gubbins, ClonalFrame predicts several smaller recombinations in accordance with its prior expectation of the geometric distribution of recombination lengths characteristic of genetic transformation (48) rather than phage movement. Hence Gubbins is effective even in cases where recombination primarily represents the movement of MGEs, with few false positives predicted elsewhere in the genome. Furthermore, this flexibility can also be considered an advantage when detecting homologous recombinations, as Gubbins has been previously shown to identify irregularly-sized ‘mosaic recombinations’ rather than the individual segments of recombinant sequence that more closely follow a geometric distribution (48). Defining the boundaries of recombinations can be an important source of error: 21% of substitutions incorrectly inferred to be point mutations in the simulation where *p*_rec_ was 0.75 shown in Figure 1 were directly adjacent to inferred recombinations, suggesting the predicted recombinations were too short. Hence identifying larger composite events reduces the probability of substitutions at the edges of sequence imports being incorrectly inferred to be point mutations.

As datasets inevitably expand in the future, the elevated density of sampling will increase the precision of ancestral sequence reconstructions by software such as Gubbins. The consequent shortening of phylogenetic branches both increases the power to detect recombination through lowering the background density of point mutations, and decreases the time over which selection has to act on any one branch to disrupt the assumed uniform distribution of these mutations, a potential source of false positives not accounted for in the simulations described here. These datasets will be computationally challenging; hence, Gubbins has been developed to have the scope for further optimization. New phylogenetic and sequence reconstruction methods can be easily incorporated. The current version can employ either FastTree, RAxML or a combination of both. FastTree and RAxML may give noticeably different results if there are long branches in the phylogeny; comparisons between the two methods can quickly be achieved by employing the hybrid approach that uses FastTree to build the initial phylogeny from which the first prediction of recombination is made, and RAxML in later iterations. Recombination predictions are generated for each branch independently, which should make this aspect of the software parallelizable as becomes necessary. It is likely that the central algorithm may also be improved with simple extensions, as at present the phylogeny is inferred only using point mutations, without drawing on the evidence of recombinations shared through common ancestry. This can be added into the software as our understanding of bacterial recombination improves. Hence Gubbins is a tool that can rapidly and accurately process currently available bacterial genome alignments, with the potential for application to large surveillance datasets in the future.

## AVAILABILITY

Gubbins is freely available for download from https://github.com/sanger-pathogens/Gubbins and http://sanger-pathogens.github.io/gubbins/.

## SUPPLEMENTARY DATA

Supplementary Data are available at NAR Online.

SUPPLEMENTARY DATA

## References

[1] Smith J.M., Smith N.H., O'Rourke M., Spratt B.G. (1993). How clonal are bacteria?. Proc. Natl Acad. Sci. U.S.A..

[2] Achtman M. (2008). Evolution, population structure, and phylogeography of genetically monomorphic bacterial pathogens. Annu. Rev. Microbiol..

[3] Holt K.E., Parkhill J., Mazzoni C.J., Roumagnac P., Weill F.X., Goodhead I., Rance R., Baker S., Maskell D.J., Wain J. (2008). High-throughput sequencing provides insights into genome variation and evolution in *Salmonella* Typhi. Nat. Genet..

[4] Harris S.R., Feil E.J., Holden M.T., Quail M.A., Nickerson E.K., Chantratita N., Gardete S., Tavares A., Day N., Lindsay J.A. (2010). Evolution of MRSA during hospital transmission and intercontinental spread. Science.

[5] Didelot X., Achtman M., Parkhill J., Thomson N.R., Falush D. (2007). A bimodal pattern of relatedness between the Salmonella Paratyphi A and Typhi genomes: convergence or divergence by homologous recombination?. Genome Res..

[6] Robinson D.A., Enright M.C. (2004). Evolution of *Staphylococcus aureus* by large chromosomal replacements. J. Bacteriol..

[7] Dubnau D. (1999). DNA uptake in bacteria. Annu. Rev. Microbiol..

[8] Croucher N.J., Harris S.R., Grad Y.H., Hanage W.P. (2013). Bacterial genomes in epidemiology—present and future. Philos. Trans. R. Soc. B Biol. Sci..

[9] Sneath P.H.A., Sackin M.J., Ambler R.P. (1975). Detecting evolutionary incompatibilities from protein sequences. Syst. Zool..

[10] Stephens J.C. (1985). Statistical methods of DNA sequence analysis: detection of intragenic recombination or gene conversion. Mol. Biol. Evol..

[11] Sawyer S. (1989). Statistical tests for detecting gene conversion. Mol. Biol. Evol..

[12] Maynard Smith J., Smith N.H. (1998). Detecting recombination from gene trees. Mol. Biol. Evol..

[13] Martin D.P., Lemey P., Lott M., Moulton V., Posada D., Lefeuvre P. (2010). RDP3: a flexible and fast computer program for analyzing recombination. Bioinformatics.

[14] Fang F., Ding J., Minin V.N., Suchard M.A., Dorman K.S. (2007). cBrother: relaxing parental tree assumptions for Bayesian recombination detection. Bioinformatics.

[15] Pond S.L.K., Posada D., Gravenor M.B., Woelk C.H., Frost S.D.W. (2006). GARD: a genetic algorithm for recombination detection. Bioinformatics.

[16] Smith J.M., Dowson C.G., Spratt B.G. (1991). Localized sex in bacteria. Nature.

[17] Maynard Smith J. (1992). Analyzing the mosaic structure of genes. J. Mol. Evol..

[18] Milkman R., Bridges M.M. (1993). Molecular evolution of the *Escherichia coli* chromosome. IV. Sequence comparisons. Genetics.

[19] Didelot X., Falush D. (2007). Inference of bacterial microevolution using multilocus sequence data. Genetics.

[20] Vos M., Didelot X. (2009). A comparison of homologous recombination rates in bacteria and archaea. ISME J..

[21] Didelot X., Lawson D., Darling A., Falush D. (2010). Inference of homologous recombination in bacteria using whole-genome sequences. Genetics.

[22] Nasser W., Beres S.B., Olsen R.J., Dean M.A., Rice K.A., Long S.W., Kristinsson K.G., Gottfredsson M., Vuopio J., Raisanen K. (2014). Evolutionary pathway to increased virulence and epidemic group A Streptococcus disease derived from 3,615 genome sequences. Proc. Natl Acad. Sci. U.S.A..

[23] Marttinen P., Hanage W.P., Croucher N.J., Connor T.R., Harris S.R., Bentley S.D., Corander J. (2012). Detection of recombination events in bacterial genomes from large population samples. Nucleic Acids Res..

[24] Martin D.P., Lemey P., Posada D. (2011). Analysing recombination in nucleotide sequences. Mol. Ecol. Resour..

[25] Croucher N.J., Hanage W.P., Harris S.R., McGee L., van der Linden M., de Lencastre H., Sá-Leão R., Song J.H., Ko K.S., Beall B. (2014). Variable recombination dynamics during the emergence, transmission and ‘disarming’ of a multidrug-resistant pneumococcal clone. BMC Biol..

[26] Croucher N.J., Harris S.R., Fraser C., Quail M.A., Burton J., van der Linden M., McGee L., von Gottberg A., Song J.H., Ko K.S. (2011). Rapid pneumococcal evolution in response to clinical interventions. Science.

[27] Croucher N.J., Mitchell A.M., Gould K.A., Inverarity D., Barquist L., Feltwell T., Fookes M.C., Harris S.R., Dordel J., Salter S.J. (2013). Dominant role of nucleotide substitution in the diversification of serotype 3 pneumococci over decades and during a single infection. PLoS Genet..

[28] Croucher N.J., Finkelstein J.A., Pelton S.I., Mitchell P.K., Lee G.M., Parkhill J., Bentley S.D., Hanage W.P., Lipsitch M. (2013). Population genomics of post-vaccine changes in pneumococcal epidemiology. Nat. Genet..

[29] Croucher N.J., Chewapreecha C., Hanage W.P., Harris S.R., McGee L., van der Linden M., Song J.H., Ko K.S., de Lencastre H., Turner C. (2014). Evidence for soft selective sweeps in the evolution of pneumococcal multidrug resistance and vaccine escape. Genome Biol. Evol..

[30] Chewapreecha C., Harris S.R., Croucher N.J., Turner C., Marttinen P., Cheng L., Pessia A., Aanensen D.M., Mather A.E., Page A.J. (2014). Dense genomic sampling identifies highways of pneumococcal recombination. Nat. Genet..

[31] Mutreja A., Kim D.W., Thomson N.R., Connor T.R., Lee J.H., Kariuki S., Croucher N.J., Choi S.Y., Harris S.R., Lebens M. (2011). Evidence for several waves of global transmission in the seventh cholera pandemic. Nature.

[32] Bryant J.M., Grogono D.M., Greaves D., Foweraker J., Roddick I., Inns T., Reacher M., Haworth C.S., Curran M.D., Harris S.R. (2013). Whole-genome sequencing to identify transmission of *Mycobacterium abscessus* between patients with cystic fibrosis: a retrospective cohort study. Lancet.

[33] Mather A.E., Reid S.W.J., Maskell D.J., Parkhill J., Fookes M.C., Harris S.R., Brown D.J., Coia J.E., Mulvey M.R., Gilmour M.W. (2013). Distinguishable epidemics of multidrug-resistant *Salmonella* Typhimurium DT104 in different hosts. Science.

[34] Okoro C.K., Kingsley R.A., Connor T.R., Harris S.R., Parry C.M., Al-Mashhadani M.N., Kariuki S., Msefula C.L., Gordon M.A., de Pinna E. (2012). Intracontinental spread of human invasive *Salmonella* Typhimurium pathovariants in sub-Saharan Africa. Nat. Genet..

[35] Bronowski C., Fookes M.C., Gilderthorp R., Ashelford K.E., Harris S.R., Phiri A., Hall N., Gordon M.A., Wain J., Hart C.A. (2013). Genomic characterisation of invasive non-typhoidal *Salmonella enterica* Subspecies enterica Serovar Bovismorbificans isolates from Malawi. PLoS Negl. Trop. Dis..

[36] Grad Y.H., Godfrey P., Cerquiera G.C., Mariani-Kurkdjian P., Gouali M., Bingen E., Shea T.P., Haas B.J., Griggs A., Young S. (2013). Comparative genomics of recent Shiga toxin-producing *Escherichia coli* O104: H4: short-term evolution of an emerging pathogen. MBio.

[37] Grad Y.H., Kirkcaldy R.D., Trees D., Dordel J., Harris S.R., Goldstein E., Weinstock H., Parkhill J., Hanage W.P., Bentley S. (2014). Genomic epidemiology of *Neisseria gonorrhoeae* with reduced susceptibility to cefixime in the USA: a retrospective observational study. Lancet Infect. Dis..

[38] Harris S.R., Clarke I.N., Seth-Smith H.M.B., Solomon A.W., Cutcliffe L.T., Marsh P., Skilton R.J., Holland M.J., Mabey D., Peeling R.W. (2012). Whole-genome analysis of diverse *Chlamydia trachomatis* strains identifies phylogenetic relationships masked by current clinical typing. Nat. Genet..

[39] Stamatakis A., Aberer A.J., Goll C., Smith S.A., Berger S.A., Izquierdo-Carrasco F. (2012). RAxML-Light: a tool for computing terabyte phylogenies. Bioinformatics.

[40] Price M.N., Dehal P.S., Arkin A.P. (2010). FastTree 2—approximately maximum-likelihood trees for large alignments. PLoS One.

[41] Ashkenazy H., Penn O., Doron-Faigenboim A., Cohen O., Cannarozzi G., Zomer O., Pupko T. (2012). FastML: a web server for probabilistic reconstruction of ancestral sequences. Nucleic Acids Res..

[42] Kulldorff M. (1997). A spatial scan statistic. Commun. Stat. Theory Methods.

[43] Husmeier D. (2005). Discriminating between rate heterogeneity and interspecific recombination in DNA sequence alignments with phylogenetic factorial hidden Markov models. Bioinformatics.

[44] Hudson R.E., Bergthorsson U., Roth J.R., Ochman H. (2002). Effect of chromosome location on bacterial mutation rates. Mol. Biol. Evol..

[45] Rice T.K., Schork N.J., Rao D.C. (2008). Methods for handling multiple testing. Adv. Genet..

[46] Mostowy R., Croucher N.J., Hanage W.P., Harris S.R., Bentley S., Fraser C. (2014). Heterogeneity in the frequency and characteristics of homologous recombination in pneumococcal evolution. PLoS Genet..

[47] Croucher N.J., Walker D., Romero P., Lennard N., Paterson G.K., Bason N.C., Mitchell A.M., Quail M.A., Andrew P.W., Parkhill J. (2009). Role of conjugative elements in the evolution of the multidrug-resistant pandemic clone *Streptococcus pneumoniae*^Spain23F^ ST81. J. Bacteriol..

[48] Croucher N.J., Harris S.R., Barquist L., Parkhill J., Bentley S.D. (2012). A high-resolution view of genome-wide pneumococcal transformation. PLoS Pathog..

[49] Castillo-Ramírez S., Corander J., Marttinen P., Aldeljawi M., Hanage W.P., Westh H., Boye K., Gulay Z., Bentley S.D., Parkhill J. (2012). Phylogeographic variation in recombination rates within a global clone of methicillin-resistant *Staphylococcus aureus* (MRSA). Genome Biol..

[50] Yahara K., Furuta Y., Oshima K., Yoshida M., Azuma T., Hattori M., Uchiyama I., Kobayashi I. (2013). Chromosome painting in silico in a bacterial species reveals fine population structure. Mol. Biol. Evol..

[51] Darling A.E., Mau B., Perna N.T. (2010). progressiveMauve: multiple genome alignment with gene gain, loss and rearrangement. PLoS One.

[52] Donati C., Hiller N.L., Tettelin H., Muzzi A., Croucher N.J., Angiuoli S.V., Oggioni M., Dunning Hotopp J.C., Hu F.Z., Riley D.R. (2010). Structure and dynamics of the pan-genome of *Streptococcus pneumoniae* and closely related species. Genome Biol..

[53] Robinson D.F., Foulds L.R. (1981). Comparison of phylogenetic trees. Math. Biosci..

[54] Bruen T.C., Philippe H., Bryant D. (2006). A simple and robust statistical test for detecting the presence of recombination. Genetics.

[55] Didelot X., Bowden R., Street T., Golubchik T., Spencer C., McVean G., Sangal V., Anjum M.F., Achtman M., Falush D. (2011). Recombination and population structure in *Salmonella enterica*. PLoS Genet..

[56] Iannelli F., Oggioni M.R., Pozzi G. (2002). Allelic variation in the highly polymorphic locus *pspC* of *Streptococcus pneumoniae*. Gene.

[57] Feil E.J., Cooper J.E., Grundmann H., Robinson D.A., Enright M.C., Berendt T., Peacock S.J., Smith J.M., Murphy M., Spratt B.G. (2003). How clonal is *Staphylococcus aureus*?. J. Bacteriol..

[58] Holden M.T.G., Lindsay J.A., Corton C., Quail M.A., Cockfield J.D., Pathak S., Batra R., Parkhill J., Bentley S.D., Edgeworth J.D. (2010). Genome sequence of a recently emerged, highly transmissible, multi-antibiotic-and antiseptic-resistant variant of methicillin-resistant *Staphylococcus aureus*, sequence type 239 (TW). J. Bacteriol..

