# Supplementary material for: Rapid phylogenetic analysis of large samples of recombinant bacterial whole genome sequences using Gubbins
Source: Nucleic Acids Res. 2014 Nov 20;43(3):e15. doi: 10.1093/nar/gku1196 (PMC4330336; doi:10.1093/nar/gku1196)
Supplement: SUPPLEMENTARY DATA [file supp_43_3_e15__index.html]

Rapid phylogenetic analysis of large samples of recombinant bacterial whole genome sequences using Gubbins — Rapid phylogenetic analysis of large samples of recombinant bacterial whole genome sequences using Gubbins — SUPPLEMENTARY DATA 

# Rapid phylogenetic analysis of large samples of recombinant bacterial whole genome sequences using Gubbins

## SUPPLEMENTARY DATA

**Files in this Data Supplement:**

- SUPPLEMENTARY DATA
