# Supplementary material for: Rapid phylogenetic analysis of large samples of recombinant bacterial whole genome sequences using Gubbins
Source: Nucleic Acids Res. 2014 Nov 20;43(3):e15. doi: 10.1093/nar/gku1196 (PMC4330336; doi:10.1093/nar/gku1196)
Supplement: SUPPLEMENTARY DATA [file supp_gku1196_nar-01822-met-k-2014-File007.pdf]

Supplementary Materials - Rapid phylogenetic analysis of large samples of recombinant bacterial whole genome sequences using Gubbins

Figure S1

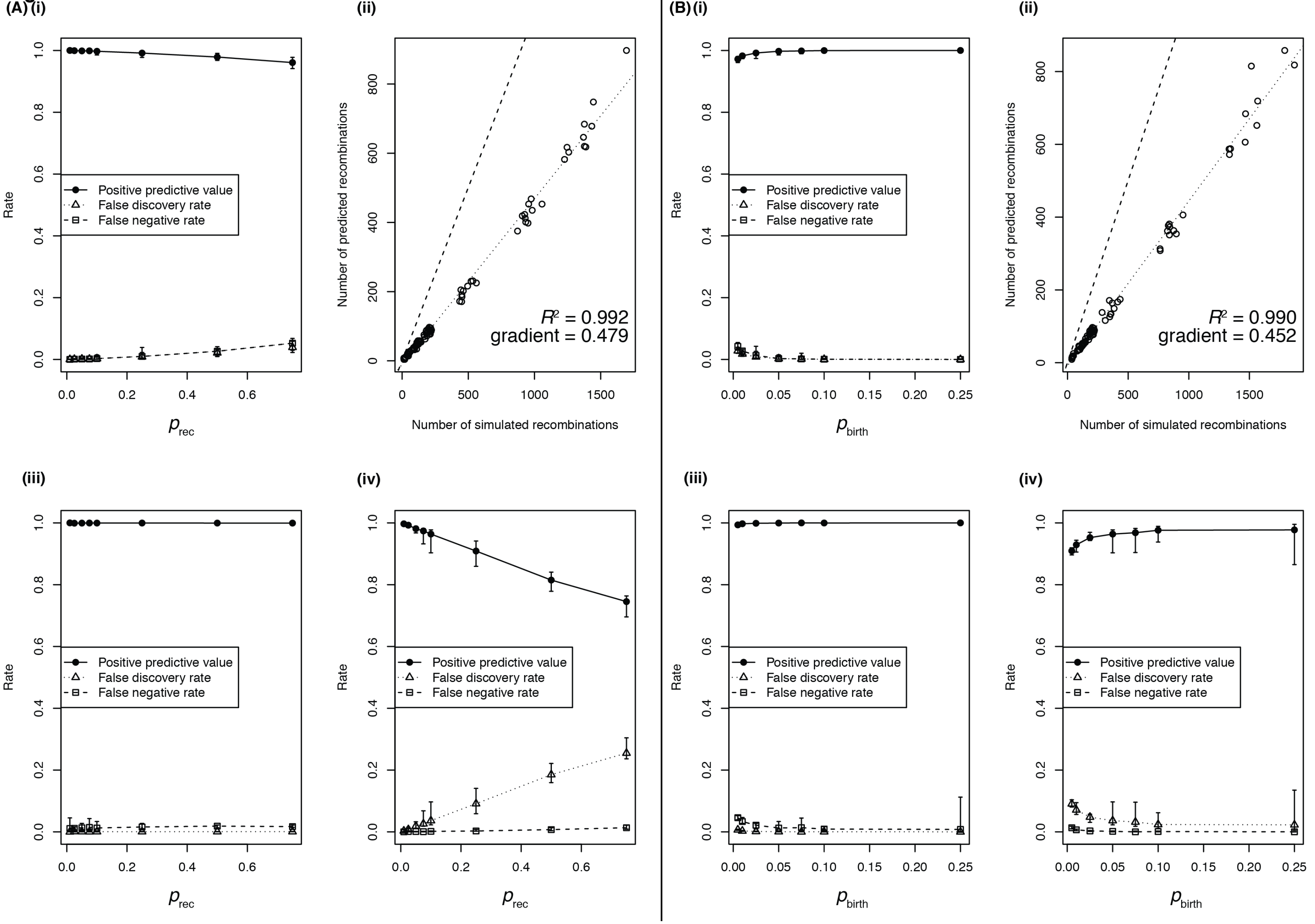

(A) (i)
Rate
Positive predictive value
False discovery rate
False negative rate
p_{rec}
(ii)
Number of predicted recombinations
Number of simulated recombinations
R^2 = 0.992
gradient = 0.479
(iii)
(iv)
(B) (i)
p_{birth}
R^2 = 0.990
gradient = 0.452

Figure S1: Accuracy of Gubbins reconstructions from simulations in which recombinations may represent imports from divergent donors or exchanges between extant sequences within the simulation. Statistics representing the impact of varying (A) p_{rec} and (B) p_{birth} are shown as in Figure 1.

Figure S2

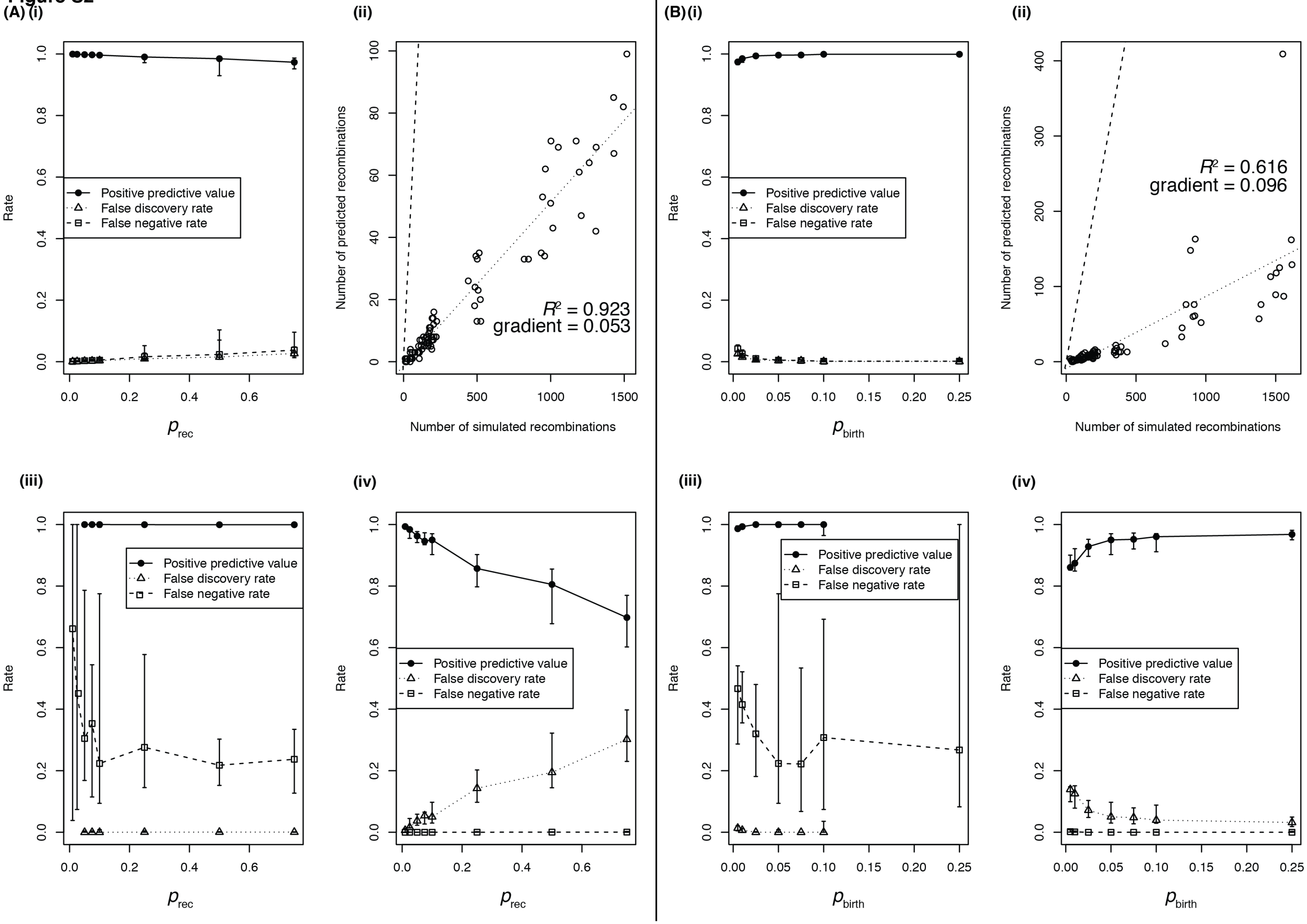

(A) (i)
Positive predictive value
False discovery rate
False negative rate
Rate
p rec
(ii)
Number of predicted recombinations
Number of simulated recombinations
R2 = 0.923
gradient = 0.053
(iii)
(iv)
(B) (i)
p birth
(ii)
R2 = 0.616
gradient = 0.096

Figure S2: Accuracy of Gubbins reconstructions from simulations using recombination donors closely related to the recipient. Statistics representing the impact of varying (A) p_{rec} and (B) p_{birth} are shown as in Figure 1. At lower values of p_{rec}, and higher values of p_{birth}, some simulations did not result in any recombinations being predicted by Gubbins, and therefore the PPV and FDR for assigning true positive base substitutions to recombinations could not be evaluated; hence these points are omitted from panels (A)(iii) and (B)(iii).

Figure S3

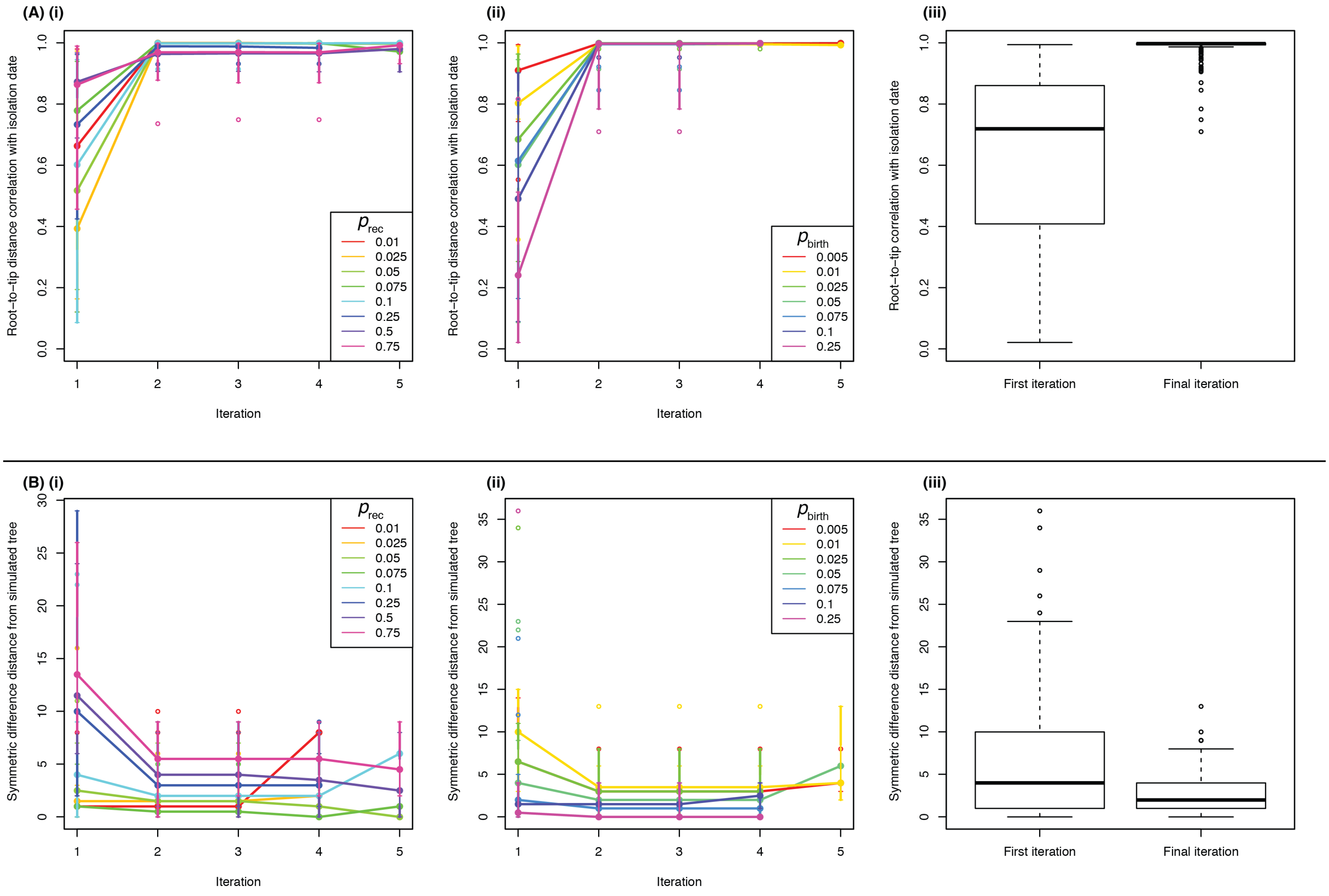

(A) (i)
Root−to−tip distance correlation with isolation date
Iteration
p_{rec}
0.01
0.025
0.05
0.075
0.1
0.25
0.5
0.75
(ii)
Root−to−tip distance correlation with isolation date
Iteration
p_{birth}
0.005
0.01
0.025
0.05
0.075
0.1
0.25
(iii)
Root−to−tip correlation with isolation date
First iteration
Final iteration
(B) (i)
Symmetric difference distance from simulated tree
Iteration
p_{rec}
0.01
0.025
0.05
0.075
0.1
0.25
0.5
0.75
(ii)
Symmetric difference distance from simulated tree
Iteration
p_{birth}
0.005
0.01
0.025
0.05
0.075
0.1
0.25
(iii)
Symmetric difference distance from simulated tree
First iteration
Final iteration

Figure S3: Accuracy of phylogenetic reconstructions from Gubbins analyses of simulations in which recombinations may represent imports from divergent donors or exchanges between extant taxa within the simulation, displayed as in Figure 2.

Figure S4

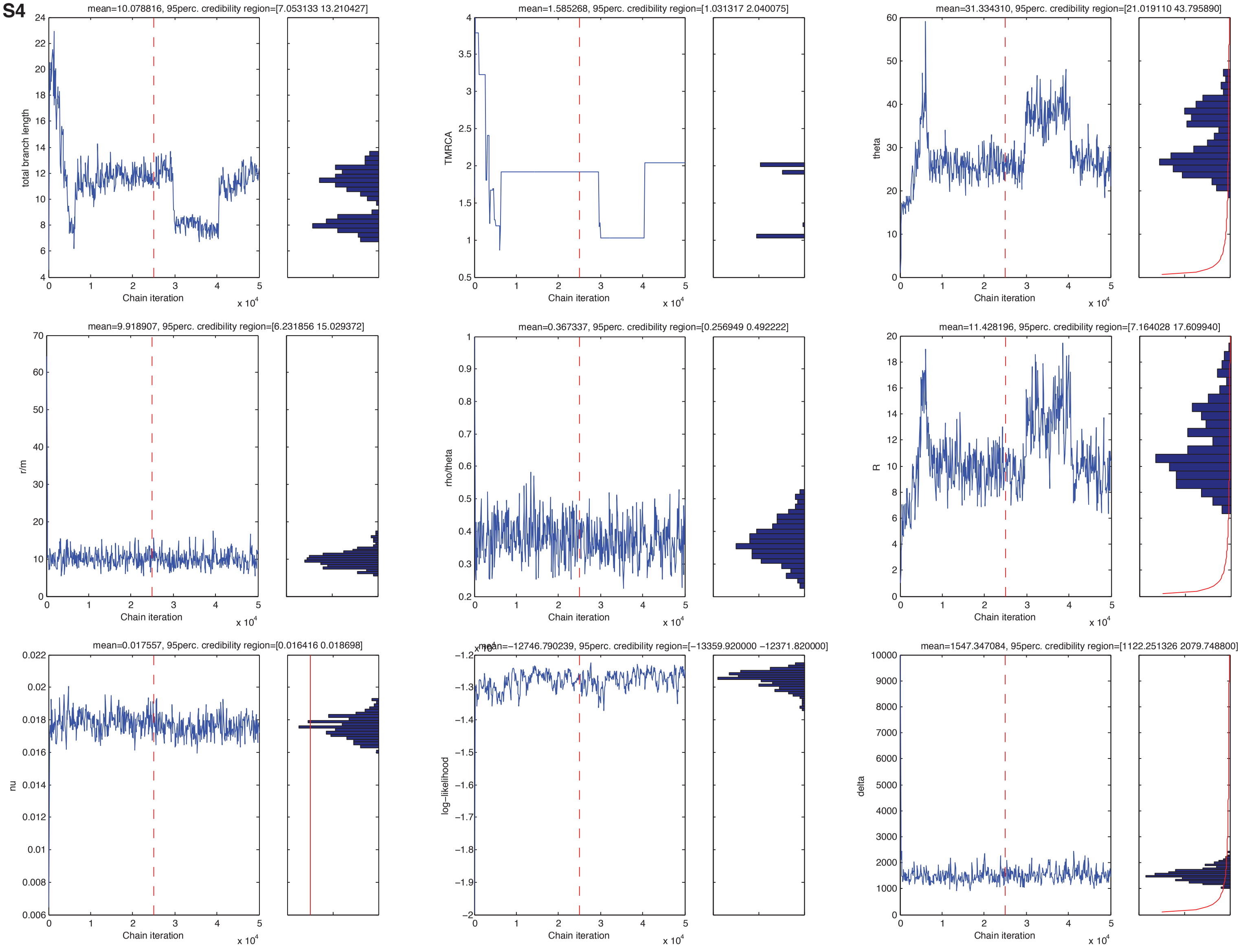
mean=10.078816, 95perc. credibility region=[7.053133 13.210427]
total branch length
Chain iteration
x 10^4
mean=1.585268, 95perc. credibility region=[1.031317 2.040075]
TMRCA
Chain iteration
x 10^4
mean=31.334310, 95perc. credibility region=[21.019110 43.795890]
theta
Chain iteration
x 10^4
mean=9.918907, 95perc. credibility region=[6.231856 15.029372]
r/m
Chain iteration
x 10^4
mean=0.367337, 95perc. credibility region=[0.256949 0.492222]
rho/theta
Chain iteration
x 10^4
mean=11.428196, 95perc. credibility region=[7.164028 17.609940]
R
Chain iteration
x 10^4
mean=0.017557, 95perc. credibility region=[0.016416 0.018698]
nu
Chain iteration
x 10^4
mean=−12746.790239, 95perc. credibility region=[−13359.920000 −12371.820000]
log−likelihood
Chain iteration
x 10^4
mean=1547.347084, 95perc. credibility region=[1122.251326 2079.748800]
delta
Chain iteration
x 10^4

Figure S4: Evidence for convergence of ClonalFrame in the analysis of *S. pneumoniae* PMEN1. The values of parameters estimated by the analysis are plotted over the duration of Monte Carlo Markov chains as blue lines, with the end of the burn-in iterations indicated by the vertical red dashed lines. The distribution of values is displayed by the histograms to the right of each plot. There is evidence of bistability in the estimates of some parameters (theta, R, TMRCA and total branch length); these all relate to the rate of mutation and recombination relative to branch length, and would not be expected to strongly impact on the comparison with the Gubbins analysis, which does not take into account the absolute length of the tree branches.

Figure S5

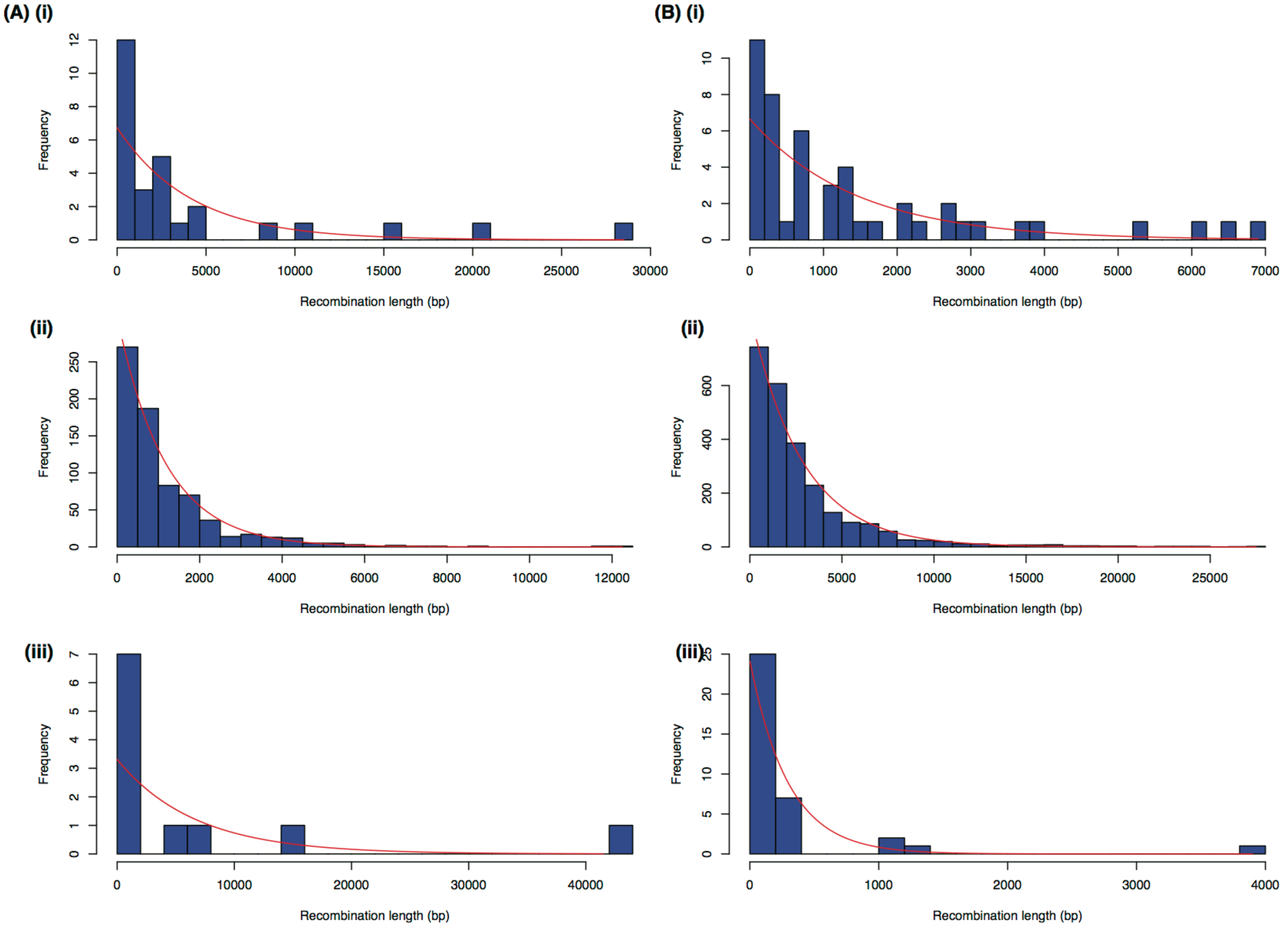
(A) (i)
Frequency
Recombination length (bp)
(ii)
Frequency
Recombination length (bp)
(iii)
Frequency
Recombination length (bp)
(B) (i)
Frequency
Recombination length (bp)
(ii)
Frequency
Recombination length (bp)
(iii)
Frequency
Recombination length (bp)

Figure S5: Predicted recombination size distributions from Gubbins and ClonalFrame. These histograms represent the lengths of the recombination events displayed in Figure 4 and Figure S6: (A) analysis performed using Gubbins on (i) *S. pneumoniae* PMEN1, (ii) *H. pylori* or (iii) *S. aureus* ST239; and (B) analysis performed using ClonalFrame on (i) *S. pneumoniae* PMEN1, (ii) *H. pylori* or (iii) *S. aureus* ST239. In each case, the red line represents the best-fitting exponential distribution.

Figure S6

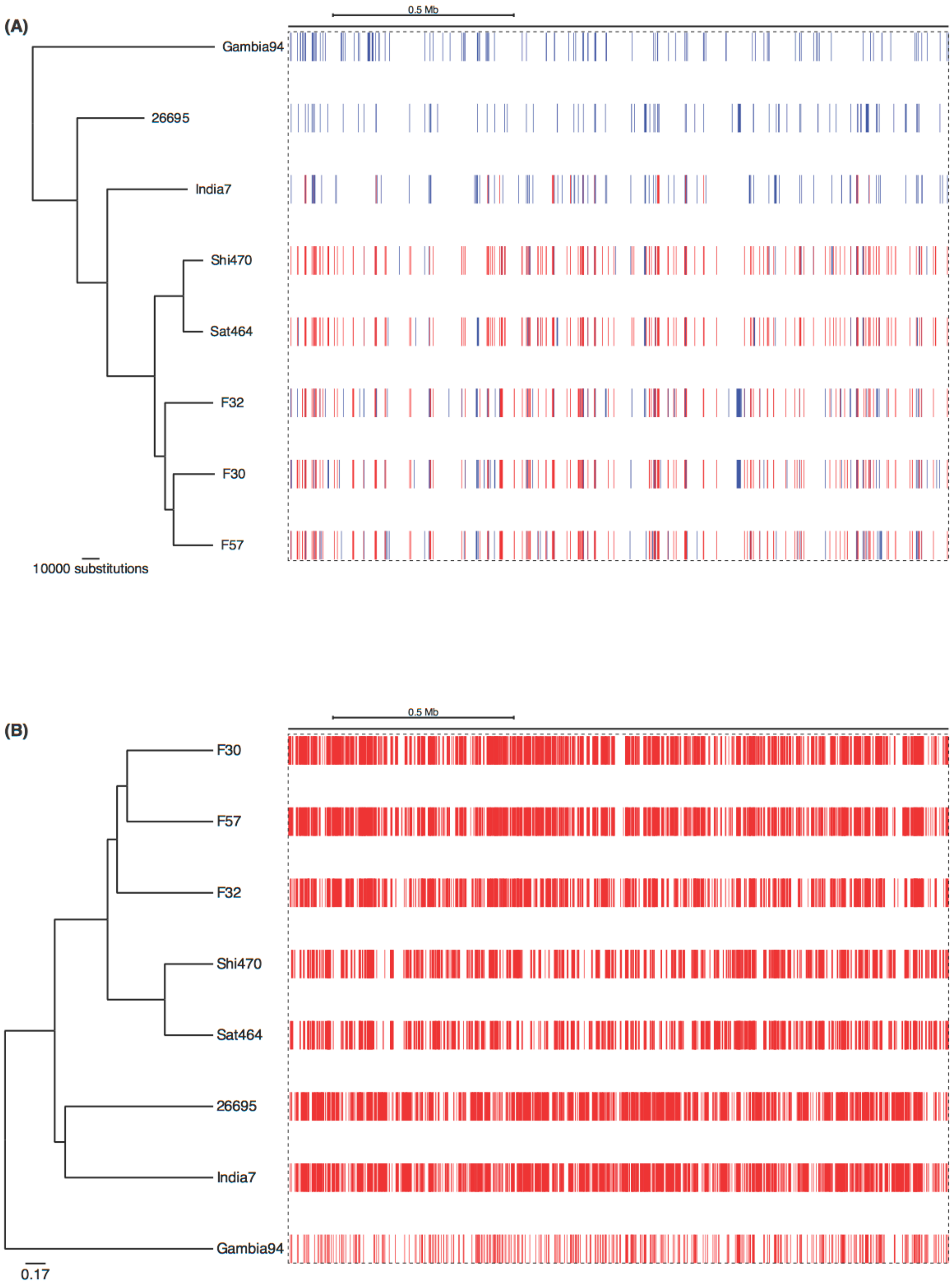
(A)
0.5 Mb
Gambia94
26695
India7
Shi470
Sat464
F32
F30
F57
10000 substitutions
(B)
0.5 Mb
F30
F57
F32
Shi470
Sat464
26695
India7
Gambia94
0.17

Figure S6: Comparison of ClonalFrame and Gubbins analyses of *H. pylori* sequences. Eight complete publically available *H. pylori* genomes were analysed with both (A) Gubbins and (B) ClonalFrame. The results are displayed as described in Figure 5.

Figure S7

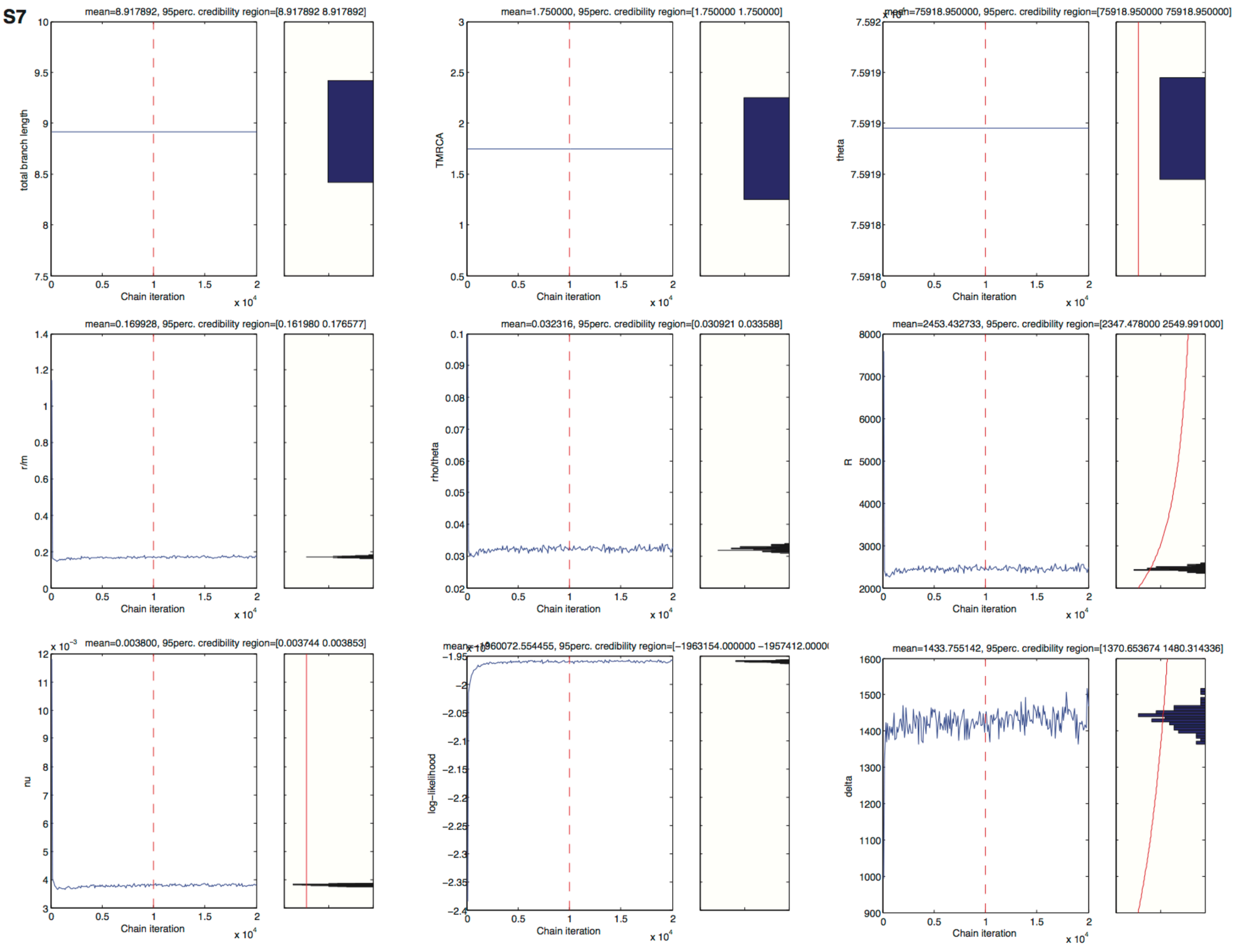
mean=8.917892, 95perc. credibility region=[8.917892 8.917892]
total branch length
mean=1.750000, 95perc. credibility region=[1.750000 1.750000]
TMRCA
mean=75918.950000, 95perc. credibility region=[75918.950000 75918.950000]
theta
mean=0.169928, 95perc. credibility region=[0.161980 0.176577]
r/m
mean=0.032316, 95perc. credibility region=[0.030921 0.033588]
rho/theta
mean=2453.432733, 95perc. credibility region=[2347.478000 2549.991000]
R
mean=0.003800, 95perc. credibility region=[0.003744 0.003853]
nu
1960072.554455, 95perc. credibility region=[−1963154.000000 −1957412.0000
log−likelihood
mean=1433.755142, 95perc. credibility region=[1370.653674 1480.314336]
delta
Chain iteration

Figure S7: Evidence for convergence of ClonalFrame in the analysis of *H. pylori*, as displayed in Figure S4. As node ages and theta were not estimated in this analysis, constraints that appeared necessary for the algorithm to converge in this case, total branch length, TMRCA and theta are invariant along the chain.

Figure S8

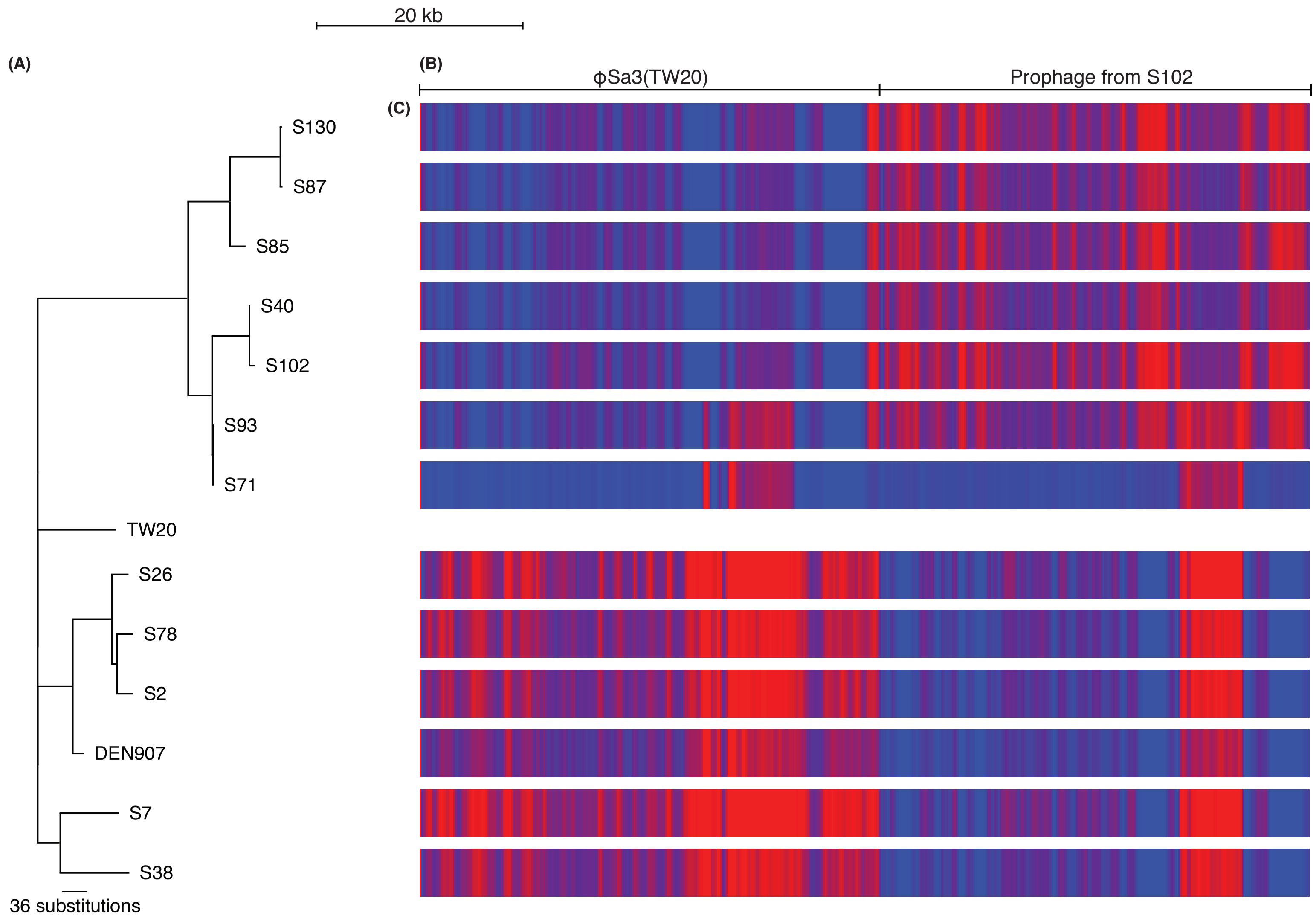

20 kb
(A)
(B)
(C)
ϕSa3(TW20)
Prophage from S102
S130
S87
S85
S40
S102
S93
S71
TW20
S26
S78
S2
DEN907
S7
S38
36 substitutions

Figure S8: Differences in prophage complement between *S. aureus* ST239 isolates. (A) The phylogeny produced by Gubbins, as displayed in Figure 5 (B)(ii). (B) Two different prophage sequences were concatenated together: that of φSa3 from TW20, and a similar prophage at the same insertion site from a *de novo* assembly of isolate S102. (C) A heatmap for each Illumina-sequenced isolate in the phylogeny represents the level of sequence read mapping across the two prophage sequences. Blue represents an absence of read mapping, red indicates high mapping, scaled to a maximum of 100-fold coverage. Read mapping was achieved using BWA as described in (25), except that only identical reads were mapped to the sequences in this instance to allow the similar viruses to be distinguished. Data were not plotted for isolate TW20, which contains φSa3, as this was the complete reference sequence against which all the other datasets were mapped.

Figure S9

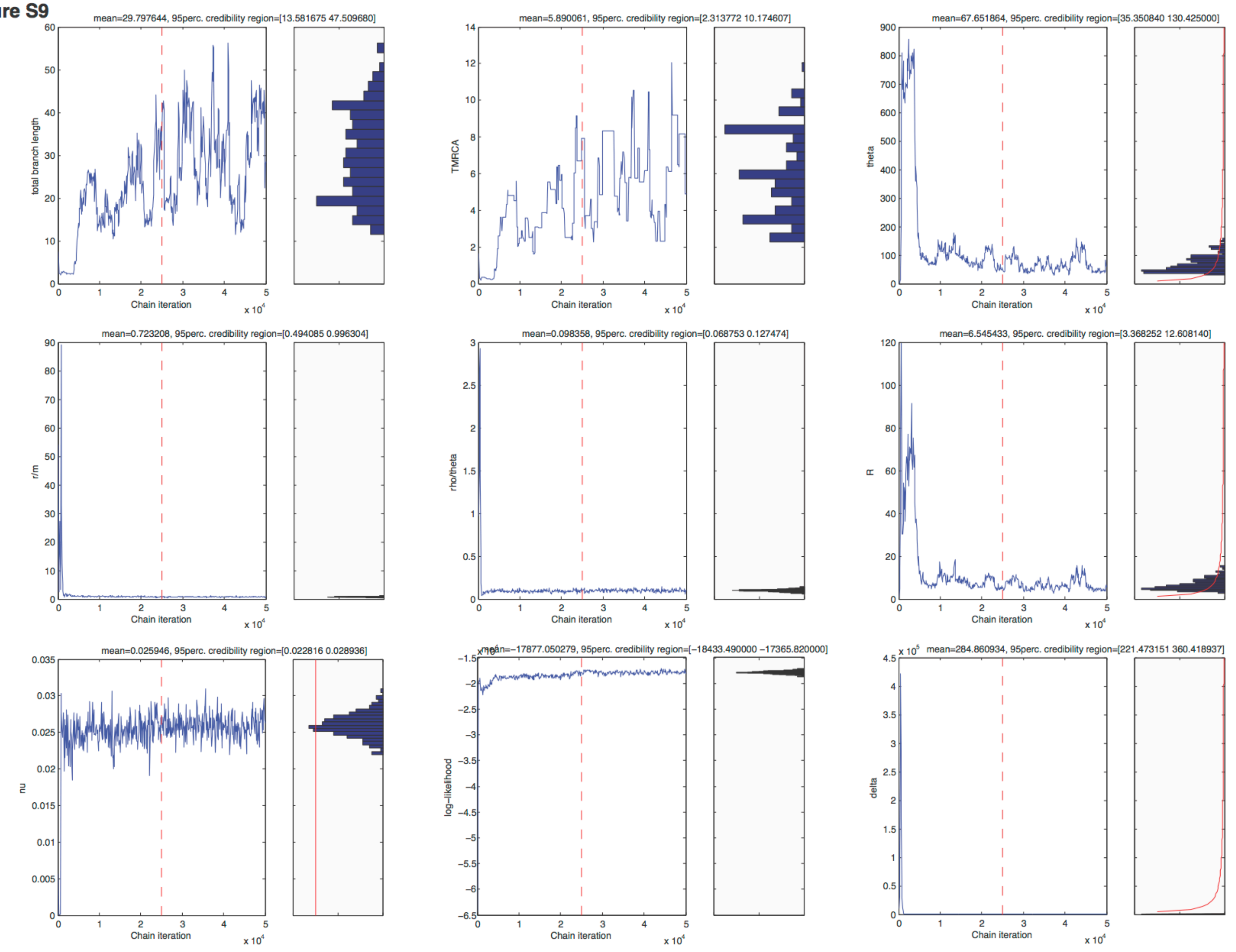

mean=29.797644, 95perc. credibility region=[13.581675 47.509680]
total branch length
Chain iteration
x 10^4
mean=5.890061, 95perc. credibility region=[2.313772 10.174607]
TMRCA
Chain iteration
x 10^4
mean=67.651864, 95perc. credibility region=[35.350840 130.425000]
theta
Chain iteration
x 10^4
mean=0.723208, 95perc. credibility region=[0.494085 0.996304]
r/m
Chain iteration
x 10^4
mean=0.098358, 95perc. credibility region=[0.068753 0.127474]
rho/theta
Chain iteration
x 10^4
mean=6.545433, 95perc. credibility region=[3.368252 12.608140]
R
Chain iteration
x 10^4
mean=0.025946, 95perc. credibility region=[0.022816 0.028936]
nu
Chain iteration
x 10^4
mean=−17877.050279, 95perc. credibility region=[−18433.490000 −17365.820000]
log−likelihood
Chain iteration
x 10^4
x 10^5 mean=284.860934, 95perc. credibility region=[221.473151 360.418937]
delta
Chain iteration
x 10^4

Figure S9: Evidence for convergence of ClonalFrame in the analysis of *S. aureus* ST239, as displayed in Figure S4.

Table S1: Details of genomes used as sequence donors in simulations analysed in Figure 1.

**Genome**	**Accession code**
Streptococcus pneumoniae 670-6B	CP002176
Streptococcus pneumoniae 70585	CP000918
Streptococcus pneumoniae AP200	CP002121
Streptococcus pneumoniae CGSP14	CP001033
Streptococcus pneumoniae D39	CP000410
Streptococcus pneumoniae G54	CP001015
Streptococcus pneumoniae gamPNI0373	CP001845
Streptococcus pneumoniae Hungary19A-6	CP000936
Streptococcus pneumoniae INV104	FQ312030
Streptococcus pneumoniae INV200	FQ312029
Streptococcus pneumoniae JJA	CP000919
Streptococcus pneumoniae OXC141	FQ312027
Streptococcus pneumoniae P1031	CP000920
Streptococcus pneumoniae R6	AE007317
Streptococcus pneumoniae ST556	CP003357
Streptococcus pneumoniae Taiwan19F-14	CP000921
Streptococcus pneumoniae TCH8431/19A	CP001993
Streptococcus pneumoniae TIGR4	AE005672
